# Phylogeography, Historical Population Demography, and Climatic Modeling of Two Bird Species Uncover Past Connections Between Amazonia and the Atlantic Forest

**DOI:** 10.1002/ece3.70587

**Published:** 2024-12-09

**Authors:** Ivandy N. Castro‐Astor, Joel Cracraft, José G. Tello, Maria Alice S. Alves, William M. Mauck, Alexandre Aleixo, Charles Duca, Ana Carolina Carnaval

**Affiliations:** ^1^ Department of Biology, City College of New York and Biology Program at CUNY Graduate Center City University of New York New York City New York USA; ^2^ Department of Ornithology American Museum of Natural History New York City New York USA; ^3^ Department of Biology Long Island University Brooklyn New York USA; ^4^ Departamento de Ecologia Universidade do Estado do Rio de Janeiro Rio de Janeiro Brazil; ^5^ IQVIA Inc Durham North Carolina USA; ^6^ Coordenação de Zoologia Museu Paraense Emílio Goeldi Belém Brazil; ^7^ Instituto Tecnológico Vale Desenvolvimento Sustentável Belém Brazil; ^8^ Universidade Vila Velha, Unidade Acadêmica II – Biomédicas Vila Velha Brazil; ^9^ Biology Ph.D. Program CUNY Graduate Center New York City New York USA

**Keywords:** Atlantic Forest, *Ceratopipra rubrocapilla*, historical demography, mitochondrial DNA, phylogeography, *Pseudopipra pipra*, species distribution modeling

## Abstract

We combined mitochondrial DNA sequence data and paleoclimatic distribution models to document phylogeographic patterns and investigate the historical demography of two manakins, *Ceratopipra rubrocapilla* and *Pseudopipra pipra*, as well as to explore connections between Amazonia and the Atlantic Forest. ND2 sequences of *C. rubrocapilla* (75 individuals, 24 sites) and *P. pipra* (196, 77) were used in Bayesian inference and maximum likelihood analyses. We estimated mitochondrial nucleotide diversity, employed statistical tests to detect deviations from neutral evolution and constant population sizes, and used species distribution modeling to infer the location of suitable climate for both species under present‐day conditions, the Last Glacial Maximum (LGM), and the Last Interglacial Maximum (LIG). Mitochondrial sequence data from *C. rubrocapilla* indicate one Amazonian and one Atlantic Forest haplogroup. In *P. pipra* , we recovered a highly supported and differentiated Atlantic Forest haplogroup embedded within a large Southern Amazonian clade. Genetic and taxonomic structure in Amazonia differs widely between these two species; older *P. pipra* has a more marked genetic structure and taxonomic differentiation relative to the younger *C. rubrocapilla*. Both species have similar genetic patterns in the Atlantic Forest. Paleoclimatic distribution models suggest connections between southwestern Amazonia and the southern Atlantic Forest during the LIG, but not between eastern Amazonia and the northeastern Atlantic Forest, as suggested by previous studies. This indicates that multiple corridors, and at different locations, may have been available over the Pliocene and Pleistocene between these two regions.

## Graphical abstract

We combined mitochondrial DNA sequence data and paleoclimatic distribution models to document phylogeographic patterns and investigate the historical demography of two manakins, *Ceratopipra rubrocapilla* and *Pseudopipra pipra*, as well as to explore connections between Amazonia and the Atlantic Forest. Genetic and taxonomic structure in Amazonia differs widely between these taxa; *P. pipra* has a more marked genetic structure, agreeing with existing subspecies delimitation. Both species have similar genetic patterns in the Atlantic Forest. Paleoclimatic distribution models indicate connections between southwestern Amazonia and the southern Atlantic Forest during the LIG, but not between eastern Amazonia and the northeastern Atlantic Forest.

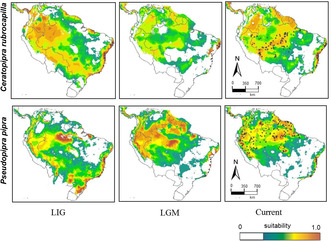

## 1 Introduction

Paleoecological and phylogeographical studies have associated Quaternary climatic oscillations with changes in the range, connectivity, and genetic structure of Neotropical Forest species (Silva 1995; Costa 2003; Oliveira‐Filho and Ratter 1995; Prates et al. 2016). Biological and geomorphological data suggest, for instance, that the Amazonian and the Atlantic coastal forests of Brazil have been connected through distinct corridors during the Quaternary, crossing the presently open and seasonally dry vegetation of the Cerrado and the Caatinga (Willis 1992; Oliveira‐Filho and Ratter 1995; Silva 1995; Oliveira, Barreto, and Suguio 1999; Auler and Smart 2001; Carnaval 2002; Costa 2003; Auler et al. 2004; Cabanne et al. 2008, 2019; Percequillo, Weksler, and Costa 2011; Weir and Price 2011; Batalha‐Filho, Fjeldså, et al. 2013; Ledo et al. 2020). Cave deposits support this claim, indicating that wetter conditions characterized at least the last two glacial periods in semiarid northeastern Brazil (Auler and Smart 2001). Palynological records also show that the Late Pleistocene climate of the presently semi‐arid São Francisco River sand dunes was more humid than present, favoring the occurrence of gallery forest (Oliveira, Barreto, and Suguio 1999). This conclusion is supported by findings of primate skeletons in the Caatinga dating back to the Late Pleistocene (Cartelle and Hartwing 1996), as well as by the presence of Amazonian and Atlantic Forest taxa in Caatinga and Cerrado biomes (Batalha‐Filho, Fjeldså, et al. 2013; Ledo et al. 2020). These changes are expected to have significantly impacted bird populations throughout the region.

Based on a comprehensive analysis of phylogenetic and geographic distribution data of 1200 species of birds, Batalha‐Filho, Fjeldså, et al. (2013) suggested three historical connections between Amazonia and Atlantic Forest: an old one (dating back to the mid‐ to late Miocene), through the southern portion of the Cerrado and the Brazilian state of Mato Grosso (see also Oliveira, Barreto, and Suguio 1999; Costa 2003; Auler et al. 2004; Wang et al. 2004; Cabanne et al. 2008; Sobral‐Souza, Lima‐Ribeiro, and Solferini 2015), and two more recent connections (in the Pliocene to Pleistocene): one through an eastern forest corridor, along the coast of the states of Maranhão, Piauí, Ceará, and Rio Grande do Norte, and another through the states of Tocantins and Bahia.

While Batalha‐Filho, Fjeldså, et al. (2013) used phylogenetic patterns to propose a general temporal and spatial model of forest connectivity, infra‐specific analyses can provide further insight on the demographic and phylogeographic history of organisms that co‐occur in these two forest domains, enhancing our understanding of the connections that enabled the colonization of the Atlantic Forest from Amazonian forms and gene flow across forest isolates. To do this, we employ mitochondrial DNA (mtDNA) sequencing and species distribution models (SDMs) to describe phylogeographic and spatial distribution patterns of two understory forest bird species broadly co‐distributed in the Atlantic Forest and Amazonia: the red‐headed manakin (*Ceratopipra rubrocapilla*) and the white‐crowned manakin (*Pseudopipra pipra*).

The monotypic species *C. rubrocapilla* occurs south of the Amazon River in Brazil, in eastern Peru, in northern and eastern Bolivia, and in the lowland Atlantic Forest (Traylor Jr. 1979; Ridgely and Tudor 1994). *Ceratopipra rubrocapilla* is sister to the southwestern Amazonian *C. chloromeros* (Ohlson, Fjeldsa, and Ericson 2013; Leite et al. 2021; but see Harvey et al. 2020). A recent study uncovered shallow population structure and supported widespread gene flow across the range of *C. rubrocapilla*, including a close relationship between Atlantic Forest and eastern Amazonian (Belém + Xingu areas of endemism) populations (Mikkelsen et al. 2024). These patterns supported extensive recent dispersal between these ecosystems, in addition to revealing that the Atlantic Forest population had the lowest genetic diversity, possibly due to the smaller size of this biome and historical bottlenecks related to a recent colonization originating in the Amazon (Mikkelsen et al. 2024).

*Pseudopipra pipra* is more broadly distributed, occurring from Costa Rica to Amazonia and the lowland Atlantic Forest (Ridgely and Tudor 1994). It is also widely variable geographically, with 13 subspecies recognized based on plumage and relative size differences (Traylor Jr. 1979). A recent genomic phylogeographic study of *P. pipra* showed a pattern of Andean origination, with subsequent diversification into the Amazonian lowlands, with the Atlantic Forest population being embedded within Southern Amazonia (Berv et al. 2021). That study also found that *Pseudopipra* constitutes a species‐complex composed of at least eight distinct taxa which have arisen in the last ~2.5 Ma. *Ceratopipra* and *Pseudopipra* are sister genera (Tello et al. 2009; Ohlson, Fjeldsa, and Ericson 2013; Leite et al. 2021).

Using sequences of the mitochondrial marker NADH dehydrogenase subunit II (ND2), we inferred intra‐specific genealogies for these two manakin species and tentatively dated the divergence times of major lineages, examined the levels of genetic structure and connectivity between the Atlantic Forest and Amazonia for these taxa, and sought for evidence of demographic changes over time. We selected this marker given that it has been successfully used to explore these questions, and because published sequences of our target and other (closely related) species are available to complement our sampling (see Section 2). Through SDMs, we also combined occurrence data with descriptors of the climate today and in the past (at 21,000 [Last Glacial Maximum—LGM] and 120,000 [Last Interglacial Maximum—LIG] years ago), inferring potential range changes of these two species over time (Elith and Leathwich 2009). Although the temporal scale of the paleoclimatic data used in our inferences of past ranges may not match the timing of forest colonization inferred by the molecular data, we explored whether the models of former species ranges provide useful insight into the spatial location of potential connections between Amazonian and Atlantic rainforests during climatic extremes of the Late Quaternary. Specifically, we examined the more recent (i.e., Pleistocene) northeastern connections between Amazonia and the Atlantic Forest (Batalha‐Filho, Fjeldså, et al. 2013), which are expected to fall within the divergence times expected across intraspecific lineages of *C. rubrocapilla* and *P. pipra*.

## 2 Materials and Methods

### 2.1 DNA Sampling and Sequencing

Tissue samples of liver or blood from 74 *C. rubrocapilla* (24 localities) and 39 *P*. *pipra* specimens (24 localities) were sequenced for this study (see Table S1 for list and GenBank accession numbers). Standard molecular methods were used to extract, isolate, amplify, and sequence the mitochondrial NADH dehydrogenase subunit II gene (ND2; 1041 bp; for an extended description of methods see File S1). Electropherograms were edited and assembled in Geneious ver. 5.5 (Biomatters, available from http://www.geneious.com/). Alignments were visually inspected. Our dataset was then augmented with published sequences of *C. rubrocapilla* (1) and *P. pipra* (157) (see Table S1), the latter being possible thanks to Berv et al. (2021) study. Sequences of *Ceratopipra chloromeros*, *C. erythrocephala* , *C. mentalis* , and *Machaeropterus deliciosus* obtained from GenBank (see Table S1) were used to root the phylogenetic tree of *C. rubrocapilla*. For the analyses of *P. pipra* , sequences of *C. rubrocapilla* (PQ272356), *Heterocercus linteatus* , and *Machaeropterus deliciosus* were used as outgroups (see Table S1).

### 2.2 Phylogenetic Analyses and Dating

Phylogenetic analyses were performed with Bayesian inference (BI) in MrBayes ver. 3.2 (Huelsenbeck and Ronquist 2001; Ronquist et al. 2012) and maximum likelihood (ML) in RAxML v8 (Stamatakis 2014). Evolutionary models for BI were selected by jModelTest v. 2.0.2 (Darriba et al. 2012), using the Akaike Information Criterion (AIC, Akaike 1973). Model fitting indicated that the GTR + I model was the best fit for the data. BI analyses used two independent runs of 10 million generations, the default temperature parameter, and default priors as starting values for the model parameters. Trees were sampled every 100 generations. Bayesian posterior probabilities were obtained from the 50% majority‐rule consensus of all trees retained after a 20% burn‐in. We used Tracer 1.6 (Rambaut et al. 2014) to ensure the log‐likelihood scores converged on similar values past the burn‐in phase. Maximum Likelihood analyses were performed using the fast approximation ML algorithm with 1000 bootstrap replicates.

Additionally, intraspecific gene genealogies were inferred using median‐joining networks using the Program PopART ver. 1.5 (Leigh and Bryant 2015, https://popart.maths.otago.ac.nz/) to facilitate visualization of the relationships among haplotypes and their geographic distributions.

Divergence time estimates were calculated with BEAST (v2.7.7; Drummond and Rambaut 2007; Heled and Drummond 2010), using an optimized relaxed clock and the same substitution models used in the BI and ML analyses. Tree calibration used the ND2 substitution rate of 2.9% sequence divergence per million years (0.0145 substitutions/site/lineage/million years; Lerner et al. 2011). We used the coalescent Skyline tree prior (Drummond et al. 2005) instead of the Yule tree prior because of its better performance in estimating divergence times for datasets with independent branch rates and a mix of sequences from species and populations (Mello et al. 2020). The substitution rate was applied with a normal distribution on the optimized relaxed molecular clock of the single partition. A total of 50 million generations were performed, sampling one tree in every 1000 generations. We examined the marginal probabilities of all samples in Tracer 1.6 (Rambaut et al. 2014) to evaluate an effective sample size (ESS) exceeding 200 for all parameters. *Manacus manacus* , *Heterocercus linteatus* , *Lepidothrix coronata* , *Machaeropterus deliciosus* , *Ceratopipra cornuta*, *C. mentalis* , *C. erythrocephala* , and *C. chloromeros* were used as outgroups (see Table S1).

### 2.3 Population Genetic Analyses

We used the major lineages recovered by the phylogenetic trees as guides to define groupings for population genetic analyses. Analyses tested for deviations from neutral evolution and constant population sizes, using summary statistics for the following population parameters: nucleotide diversity (*ᴨ*), haplotype diversity (*h*), Tajima's *D* statistic (Tajima 1989), Fu's *F*s statistic (Fu 1997), and Ramos‐Onsins and Rozas (2002) statistic. Analyses were performed in DNASP v6 (Rozas et al. 2017) using significance estimates based on 1000 coalescent simulations. For *C. rubrocapilla*, nucleotide diversity and population size changes were evaluated within the Atlantic Forest and Amazonia, excluding sample KF228555. For *P. pipra* , nucleotide diversity and population size changes were evaluated within the Atlantic Forest, in Southern Amazonia (which includes samples from south of the Amazon River [excluding Inambari]), in Northeastern Amazonia (including samples from north of the Amazon River in Suriname and Amapá in Brazil), in Northern Amazonia (including samples north of the Amazon River in Guyana and Eastern Napo in Brazil), and for Southwestern Amazonia (Inambari). For *P. pipra* , we subdivided the Atlantic Forest into a northern (north of the São Francisco River), central (between the São Francisco River and the Doce River), and southern (south of the Doce River) region.

Historical fluctuations of population size were reconstructed with extended Bayesian skyline plots (EBSPs) in BEAST, version 2.7.7 (Heled and Drummond 2010). For these analyses, we used 10,000,000 generations and an optimized relaxed lognormal molecular clock with a substitution rate of 2.9% sequence diverge per million years (Lerner et al. 2011). We used jModelTest v. 2.0.2 to select the best‐fit model of substitution (HKY) and the convergence was implemented in TRACER v.1.6.

### 2.4 Climatic Modeling

We used SDMs to infer the location of suitable areas for *C. rubrocapilla* and *P. pipra* under present‐day conditions that were then projected to two past climates: the LGM—21 ka, and the LIG—120 ka. These models were also used to evaluate possible historical connections between Amazonia and Atlantic Forest. To run the models, we used independent tests of regularization multipliers and feature classes using space Jackknife in SDMtoolbox v1.1b (Brown 2014), using MaxEnt v. 3.3.3e (Phillips, Anderson, and Schapire 2006). MaxEnt models are built from presence and background data and perform well relative to other distribution modeling approaches (Elith, Graham, and Anderson 2006).

Species occurrence records for *C. rubrocapilla* and *P. pipra* (Table S2), were obtained from museum collections (MPEG, MZUSP, MZFS, FMNH, AMNH), GBIF (http://www.gbif.org/) and online databases Xeno‐canto (http://www.xeno‐canto.org/), vetted for accuracy, and added to records from our own fieldwork. To reduce misleading spatial autocorrelation (due to biased sampling; Boria et al. 2014; Hijmans 2012; Veloz 2009), we filtered occurrence records that were less than 10 km apart from each other in ArcGIS version 10.2.1, using SDMtoolbox v1.1b (Brown 2014). This resulted in 96 records for *P. rubrocapilla* and 164 for *P. pipra* that were used for the SDM analyses.

To evaluate the performance of spatially segregated localities, we split the landscape into four regions and calibrated our models using one evaluation record and *k*−1 calibration records (where *k* is the total number of occurrence records). Because model performance can be affected by the extent of the background sampling area (Anderson 2012; Anderson and Raza 2010), model feature class, and regularization multipliers (Phillips and Dudík 2008; Shcheglovitova and Anderson 2013; Radosavljevic and Anderson 2014), we implemented best practices into our modeling exercise. To avoid overprediction, we restricted the background area to a minimum convex polygon based on the extent of the training region (i.e., based on known occurrence data), buffered by 100 km. This method avoids the prediction of suitable habitat outside of the training region. We evaluated model fit based on the lowest test omission rate (according to the 10‐percentile training presence threshold of MaxEnt; Pearson et al. 2007) and the highest value of the area under the curve (AUC) of the receiver operating characteristic plot obtained for the test data within the calibration region (Fielding and Bell 1997). We used all possible different combinations of feature classes; for each combination, we applied seven regularization multipliers (ranging from 1 to 4 at 0.5 increments). Multiple feature classes and regularization multipliers were assessed to select the setting that produced the best performing models (optimal complexity), that is, models with the least degree of overfitting and the highest discriminatory ability (Phillips and Dudík 2008; Shcheglovitova and Anderson 2013). We used the logistic default output format for model suitability values, which depicts the probability of presence (ranging from 0 to 1) based on the assumption that grid cells with locality records have a probability of presence of 0.5 (Phillips and Dudík 2008; but see Royle et al. 2012 and Hastie and Fithian 2013 for a critical review). When the models required extrapolation into no‐analog conditions, we chose to “clamp” the species' response surface, assigning the suitability level observed at the point of truncation to all grid cells in which the environmental conditions were different from those of the training points (Anderson 2013).

All SDMs were generated at 30 arc‐second resolution (~1 km^2^ near the equator), using the 19 bioclimatic variables available through WorldClim (Hijmans et al. 2005). These SDMs were then applied to paleoclimatic models by the community climate system model (CCSM) for 21 and 120 ka (http://www.ccsm.ucar.edu/; Kiehl and Gent 2004).

## 3 Results

### 3.1 Phylogenetic Analyses

**FIGURE 1:**
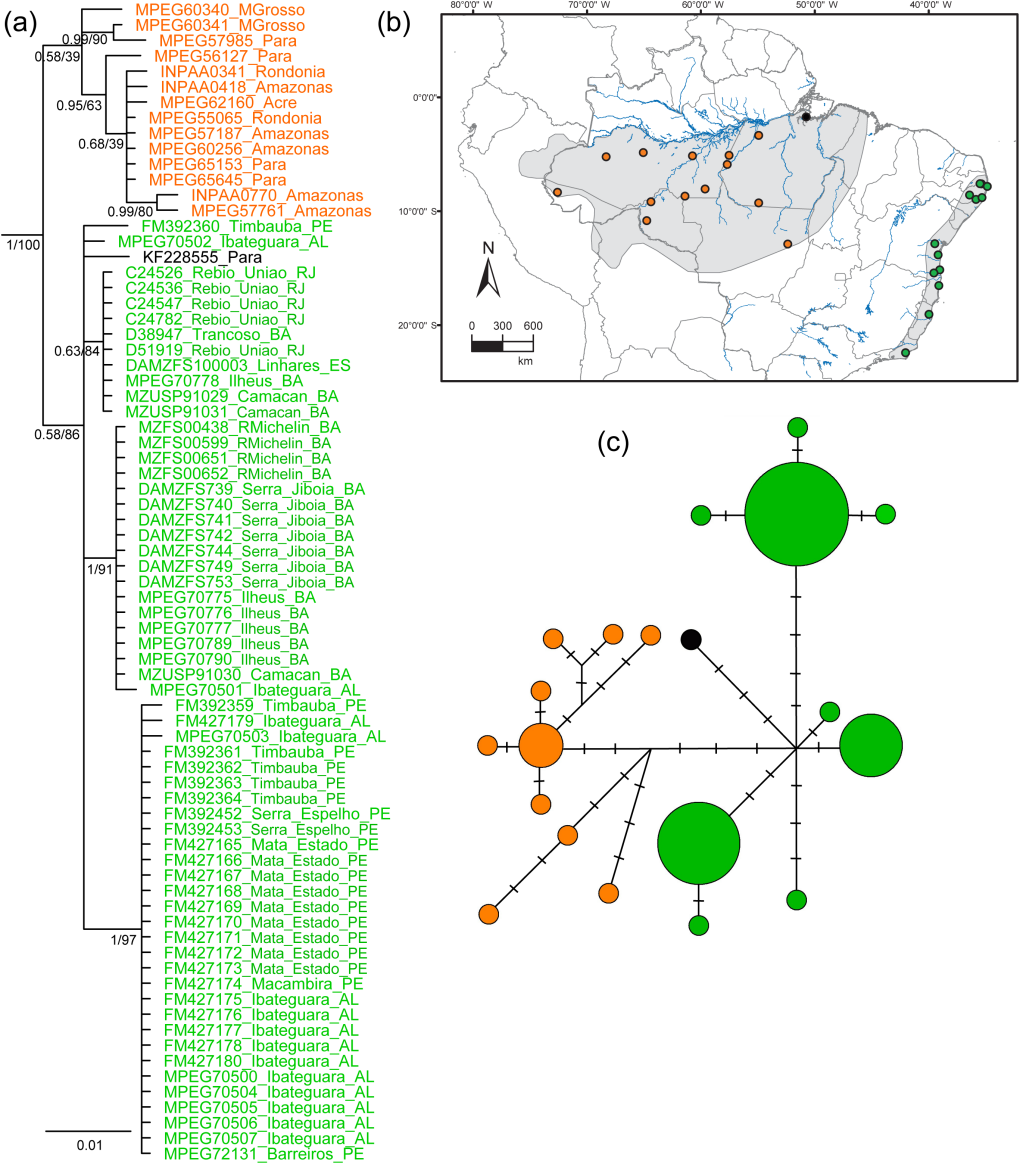
(a) Bayesian Inference of *Ceratopipra rubrocapilla* based on 1041 bp of the ND2 gene. Numbers correspond to posterior probabilities and Maximum Likelihood bootstraps. *Ceratopipra chloromeros*, *C. erythrocephala* , *C. mentalis* , and *Machaeropterus deliciosus* were used as outgroups (not shown). (b) Map showing current distribution of *C. rubrocapilla* and sampling localities included in the molecular analyses. Haplotypes are color‐coded according to localities, as shown on the map. (c) Median joining network showing all samples of *C. rubrocapilla* (1041 bp of ND2, *n* = 75 sequences). Each circle represents a haplotype, and the size of the circle is proportional to the number of individuals having that haplotype.

**FIGURE 2:**
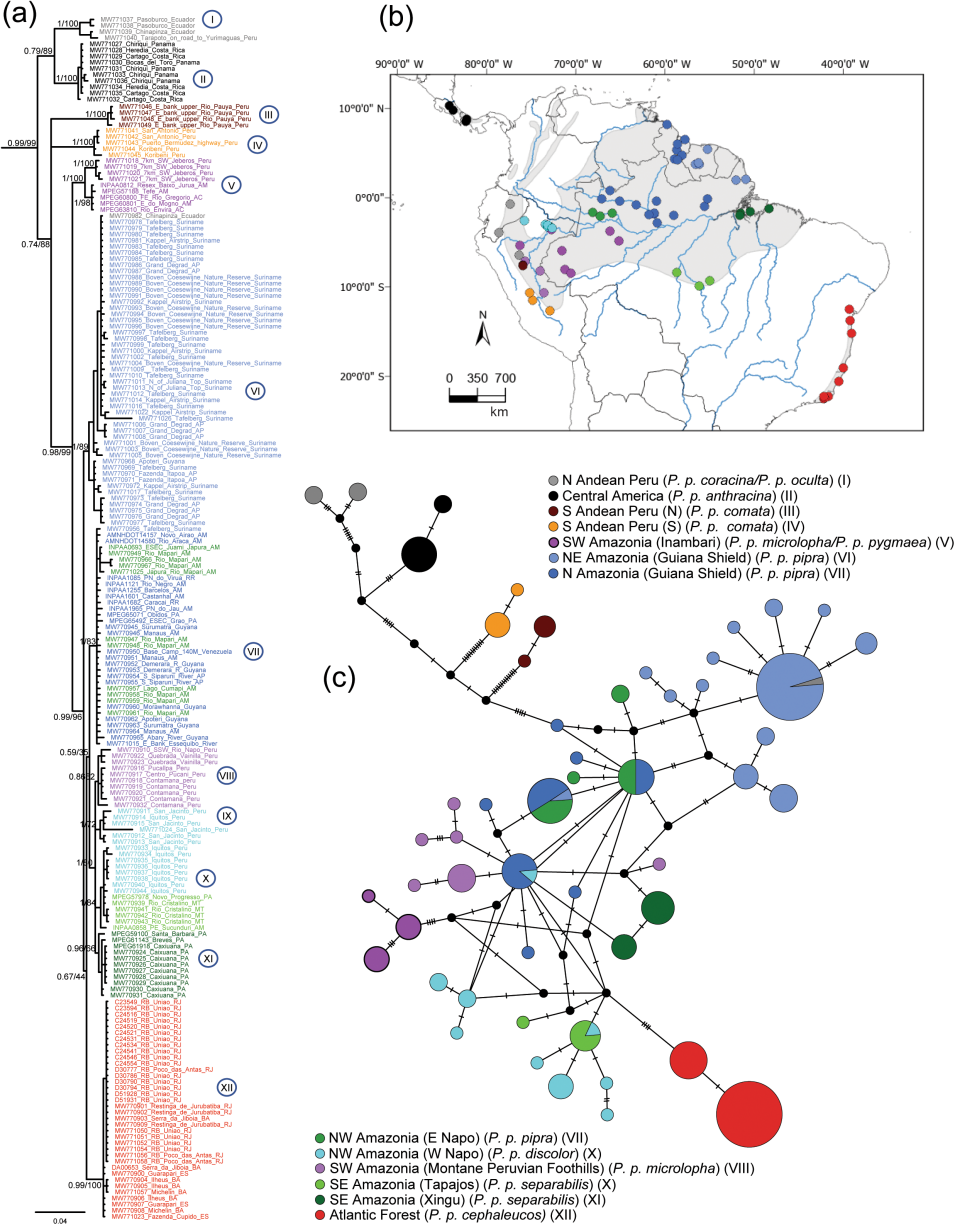
(a) Bayesian Inference of *Pseudopipra pipra* based on 1041 bp of the ND2 gene. Numbers correspond to posterior probabilities and Maximum Likelihood bootstraps. *Ceratopipra rubrocapilla*, *Heterocercus linteatus* , and *Machaeropterus deliciosus* were used as outgroups (not shown). (b) Map showing current distribution of *P. pipra* and sampling localities included in the molecular analyses. Haplotypes are color‐coded according to localities from different regions throughout the distribution, as shown on the map. (c) Median joining network showing all samples of *P. pipra* (above, 1041 bp of ND2, *n* = 196 sequences). Each circle represents a haplotype, and the size of the circle is proportional to the number of individuals having that haplotype.

Out of the 1041 bp of the ND2 gene, 37 sites were polymorphic in *C. rubrocapilla*, and 87 were polymorphic in *P. pipra* . The phylogenetic analyses of *C. rubrocapilla* uncovered two weakly supported clades, one distributed in the Atlantic Forest (Bayesian posterior probability [PP] = 0.58/ML bootstrap = 86%), and the other in Amazonia (0.58/39%). One individual (KF22855) collected in Pará (SE Amazonia) was nested within the group that included all Atlantic Forest samples (Figure 1a,b). The haplotype network uncovered 20 unique haplotypes and was consistent with the BI and ML analyses (Figure 1c). The phylogenetic analyses of *P. pipra* showed significant genetic structure with 12 well‐supported clades (Figure 2a,b): (I) N Andean Peru (1.00/100%); (II) Central America (1/100%); (III) S Andean Peru (N) (1/100%); (IV) S Andean Peru (S) (1/100%); (V) SW Amazonia (Inambari) (1/100%); (VI) NE Amazonia (Guiana Shield) (1/89%); (VII) N Amazonia (Guiana Shield)/NW Amazonia (E Napo) (1/83%); (VIII) SW Amazonia (Montane Peruvian Foothills) (0.86/62%); (IX) NW Amazonia (W Napo) (1/72%); (X) SE Amazonia (Xingu) (0.96/66%); (XI) NW Amazonia (W Napo)/SE Amazonia (Tapajos) (1/84%); and (XII) Atlantic Forest (0.99/100%). The phylogenetic trees (both BI and ML) supported a N Andean Peru‐Central American clade (0.79/89%) basal to a larger clade (0.74/88%) that includes all other lineages (Figure 2a). In this later clade, the two S Andean Peru lineages are external to a large clade (0.98/99%), where SW Amazonia (Inambari) is sister to the rest of the *P. pipra* lineages. This later clade (0.99/96%) includes six Amazonian lineages and the Atlantic Forest lineage. Relationships within this clade are mainly unresolved, except for a clade formed by the Montane Peruvian Foothills lineage from SW Amazonian (clade VIII) and three S Amazonian lineages (clades IX, X, and XI). The (BI and ML) trees also placed some specimens in unexpected clades, which may result from lack of lineage sorting or introgression. For example, one individual (MW770982) collected in Zamora Chinchipe, Ecuador, expected to belong to Clade I (N Andean Peru), was nested in the NE Amazonian Clade (VI). Additionally, the E Napo specimens (NW Amazonia) were nested in the N Amazonian Clade (VII), and some W Napo (NW Amazonia) specimens clustered with Tapajos specimens (SE Amazonia) in clade X (Figure 2a).

For this species ( *P. pipra* ), the haplotype network uncovered 52 unique haplotypes (Figure 2c), and the median‐joining network analysis revealed six distinctive groups that corresponded to N Andean Peru, Central America, S Andean Peru (N), S Andean Peru (S), SW Amazonia (Inambari), and the Atlantic Forest clades. The rest of the Amazonian clades (VI–XI) were not clearly differentiated in the haplotype network.

### 3.2 Divergence Time Estimation

**FIGURE 3:**
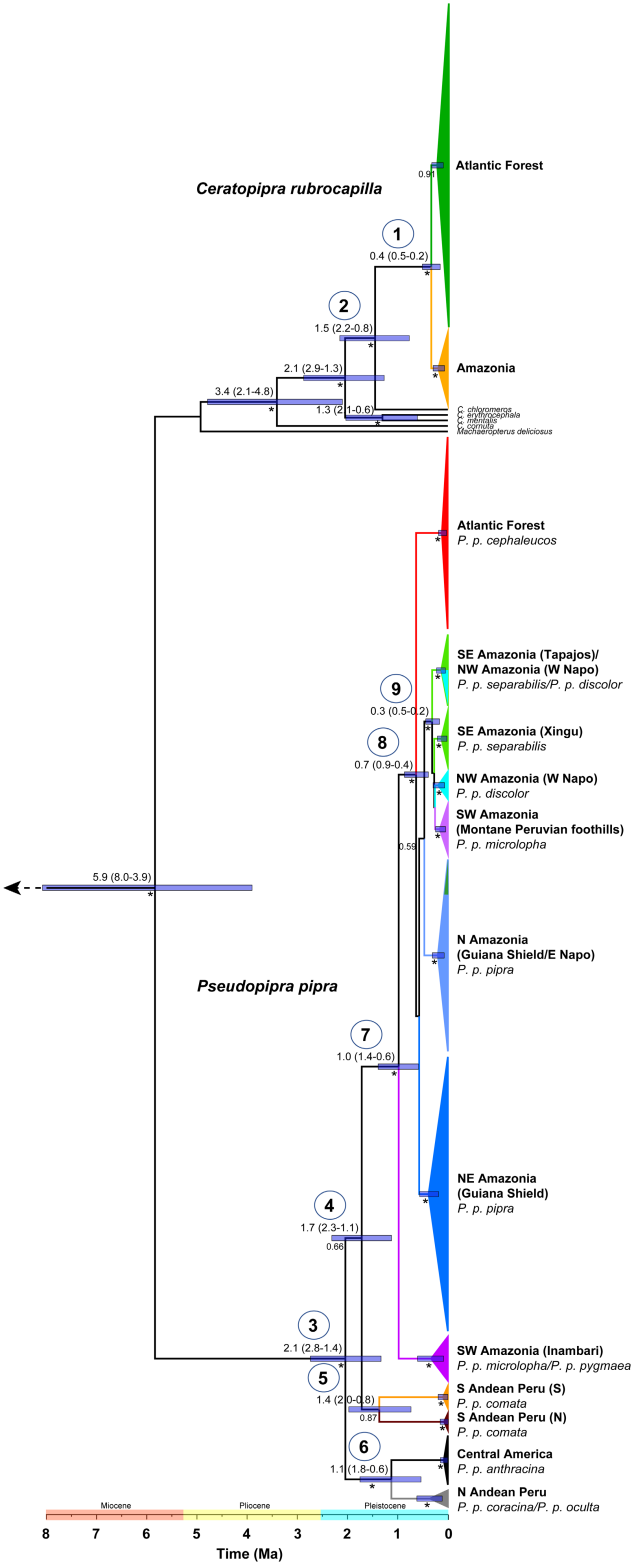
Bayesian Inference‐based divergence times of *Ceratopipra rubrocapilla* and *Pseudopipra pipra*, based on 1041 bp of the ND2 gene. *Manacus manacus* , *Heterocercus linteatus* , *Lepidothrix coronata* , *Machaeropterus deliciosus* , *Ceratopipra cornuta*, *C. mentalis* , *C. erythrocephala* , and *C. chloromeros* were used as outgroups (the first three outgroups are not showing in the tree). Numbers below the line indicate node posterior probabilities ≥ 0.5, with the * representing ≥ 0.95 posterior probability. Blue bars on nodes correspond to the 95% highest posterior density (HPD) intervals of the time estimates. For relevant nodes, numbers above the line indicate median values and 95% posterior age intervals in million years.

According to the Bayesian ultrametric tree, which was calibrated with a substitution rate of 2.9% sequence divergence per million years (Figure 3), the root age (Node 2) of *C. rubrocapilla* falls in the Early Pleistocene, between 2.2 and 0.8 Ma (Mean = 1.5 Ma). The separation of the Amazonian and Atlantic Forest lineages (Node 1) is estimated to have occurred in the Middle Pleistocene, between 0.5 and 0.2 Ma (Mean = 0.4 Ma).

The ultrametric tree resolved a clade formed by *Pseudopipra*, *Machaeropterus*, and *Ceratopipra* with a root age that falls in the Middle Miocene to Early Pliocene, between 8.0 to 3.9 Ma (Mean = 5.9 Ma) (Figure 3). In this tree, *Pseudopipra* is recovered as sister to a clade formed by *Machaeropterus* and *Ceratopipra*, but this later clade has low support (< 0.5). As such, the root age for this lineage could not be estimated. Major splits among *P. pipra* clades (Nodes 3–10) were estimated to have occurred between the Late Pliocene to Middle Pleistocene (2.8–0.2 Ma). These included the separation of the N Andean Peru‐Central American clade from a major clade that includes the rest of the *P. pipra* lineages (Node 3, 2.8–1.4 Ma, mean = 2.1 Ma). Within this latter clade, a South Andean Peru clade (Node 5) diverged first from the rest of the *P. pipra* lineages in the Early Pleistocene (Node 4, 2.3–1.1 Ma, mean = 1.7 Ma). The separation of the southern and northern portions of the S Andean Peru lineage was estimated to occur in the Early Pleistocene (2.0–0.8 Ma, mean = 1.4 Ma). The separation of the N Andean Peru and Central American lineages was placed in the Early to Middle Pleistocene (Node 6, 1.8–0.6 Ma, mean = 1.1 Ma). Subsequent splitting (Node 7) took place in the Early to Middle Pleistocene (1.4–0.6 Ma, mean = 1.0 Ma) and included the separation of SW Amazonia (Inambari) from the ancestor of a large Amazonian‐Atlantic Forest clade (Node 8). The root age of this later clade falls in the Early to Middle Pleistocene (0.9–0.4 Ma, mean = 0.7 Ma), but internal relationships among the Amazonian and Atlantic Forest lineages were not completely resolved, except for one clade that includes S and NW Amazonian lineages (Node 9). The age of this later node was estimated to occur in the Middle Pleistocene (0.5–0.3 Ma, mean 0.3 Ma). The time of the origin of the Atlantic Forest clade was not resolved but expected to occur between the estimated ages of Nodes 8 and 9 (i.e., Early to Middle Pleistocene).

### 3.3 Population Genetic Patterns and Structure Tests

In *C. rubrocapilla*, nucleotide diversity (*π*) was similar between the Atlantic Forest (0.00330) and Amazonia (0.00339; Table 1). In the Atlantic Forest, the highest diversity was found in the northern portion of the species range (0.00124) relative to the central corridor (0.00106) and southern areas (0.00000). In *P. pipra* , nucleotide diversity was higher in the Amazon (0.01118) relative to the Atlantic Forest (0.00052; Table 1). Within Amazonia, the southern haplogroup showed highest diversity (0.00564), followed by the southwestern haplogroup (0.00528), the northeastern haplogroup (0.00473), and the northern haplogroup (0.00328; Table 1). The high nucleotide diversity observed in southern Amazonia might be due to geographic structure, given that the phylogenetic analyses showed the local existence of four highly supported subgroups (distributed in the Montane Peruvian Foothills, W Napo, Xingu, and Tapajos).

**TABLE 1:** Mitochondrial NADH dehydrogenase subunit 2 (ND2) nucleotide diversity (*π*), Tajima's *D*, Fu's *F*s, and Ramos‐Onsins and Rozas *R*2 statistics for *Ceratopipra rubrocapilla* and *Pseudopipra pipra*.

Groups	*N*	Ha	*π* (SD)	Tajima's *D*		Fu's *F*s		*R*2	
				Obs.	*p*	Obs.	*p*	Obs.	*p*
*Ceratopipra rubrocapilla*									
Atlantic Forest	60	9	0.00330 (0.00014)	0.204	0.651	1.255	0.740	0.110	0.597
Northern	33	7	0.00124 (0.00053)	−2.051	0.002*	−1.708	0.153	0.059	0.019*
Central	22	2	0.00106 (0.00029)	0.883	0.817	3.332	0.955	0.184	0.783
Southern	5	1	0.00000 (0.00000)	—	—	—	—	—	—
Amazonia	14	10	0.00339 (0.00071)	−1.403	0.069	−3.872	0.021*	0.077	0.000*
*Pseudopipra pipra*									
Atlantic Forest	36	3	0.00052 (0.00013)	0.244	0.640	0.346	0.569	0.136	0.518
Central	08	3	0.00075 (0.00019)	0.069	0.557	−0.224	0.277	0.213	0.238
Southern	28	2	0.00013 (0.00008)	−0.741	0.117	−0.380	0.368	0.069	0.000*
Amazonia	136	42	0.01118 (0.00055)	−1.486	0.036*	−22.096	0.000*	0.047	0.067
Southern Amazonia	41	23	0.00564 (0.00070)	−1.780	0.010*	−8.594	0.004*	0.062	0.040*
Northeastern Amazonia (Guiana Shield)	50	13	0.00473 (0.00061)	−1.105	0.123	−3.496	0.071	0.068	0.134
Northern Amazonia (Guiana Shield/E Napo)	36	11	0.00328 (0.00038)	−1.370	0.082	−5.353	0.001*	0.061	0.025*
SW Amazonia (Inambari)	9	6	0.00528 (0.00084)	1.164	0.894	0.208	0.523	0.205	0.802

Abbreviations: Ha, number of haplotypes; *N*, number of individuals; Obs., observed value; SD, standard deviation.

* Significant values.

**FIGURE 4:**
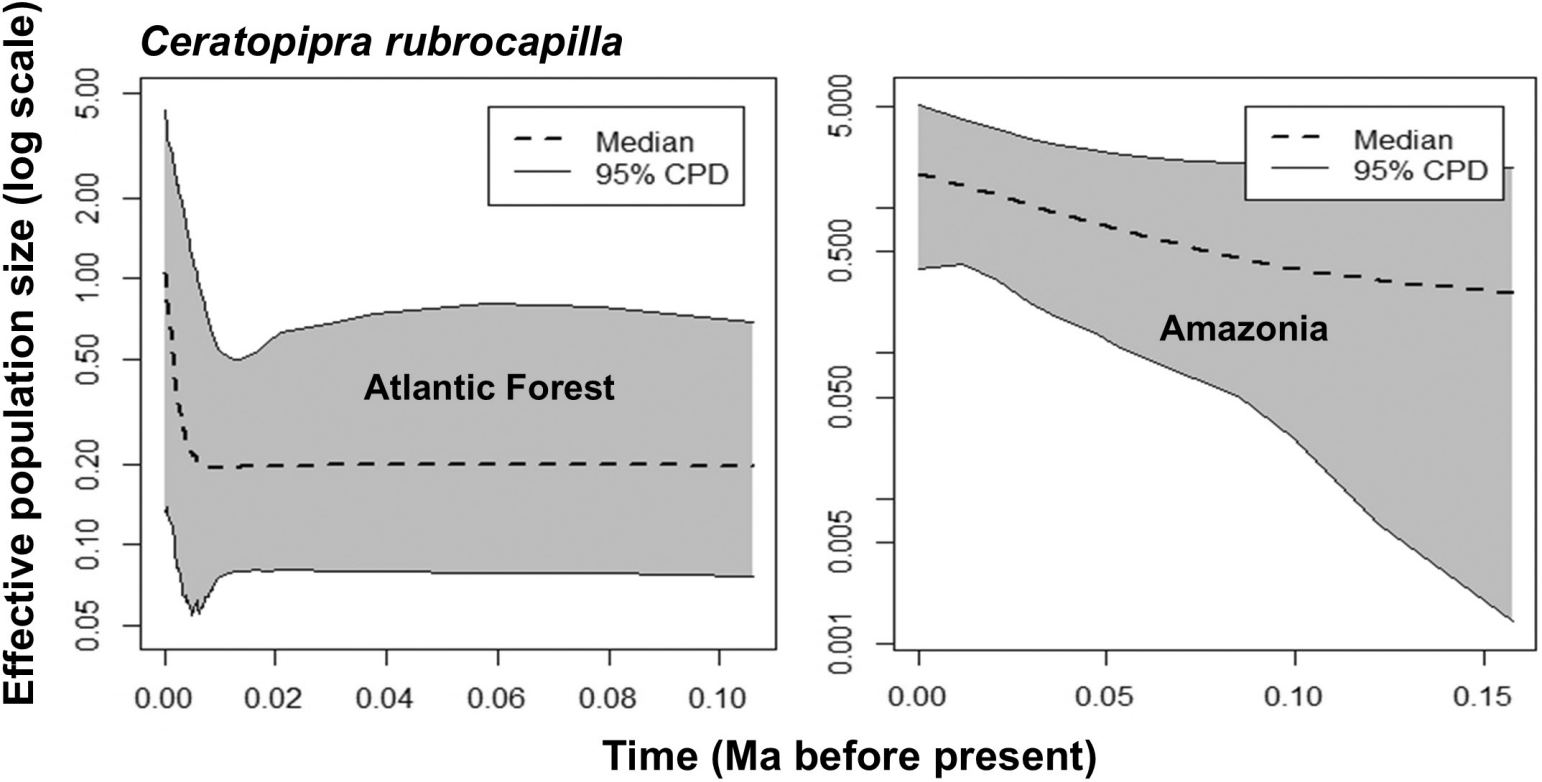
Demographic history of *Ceratopipra rubrocapilla* inferred through extended Bayesian skyline plots (EBSPs) estimated in BEAST, based on ND2 sequence data. Dashed lines depict median population size, area in gray denotes 95% Bayesian Credible Intervals. Time scale in millions of years before present.

**FIGURE 5:**
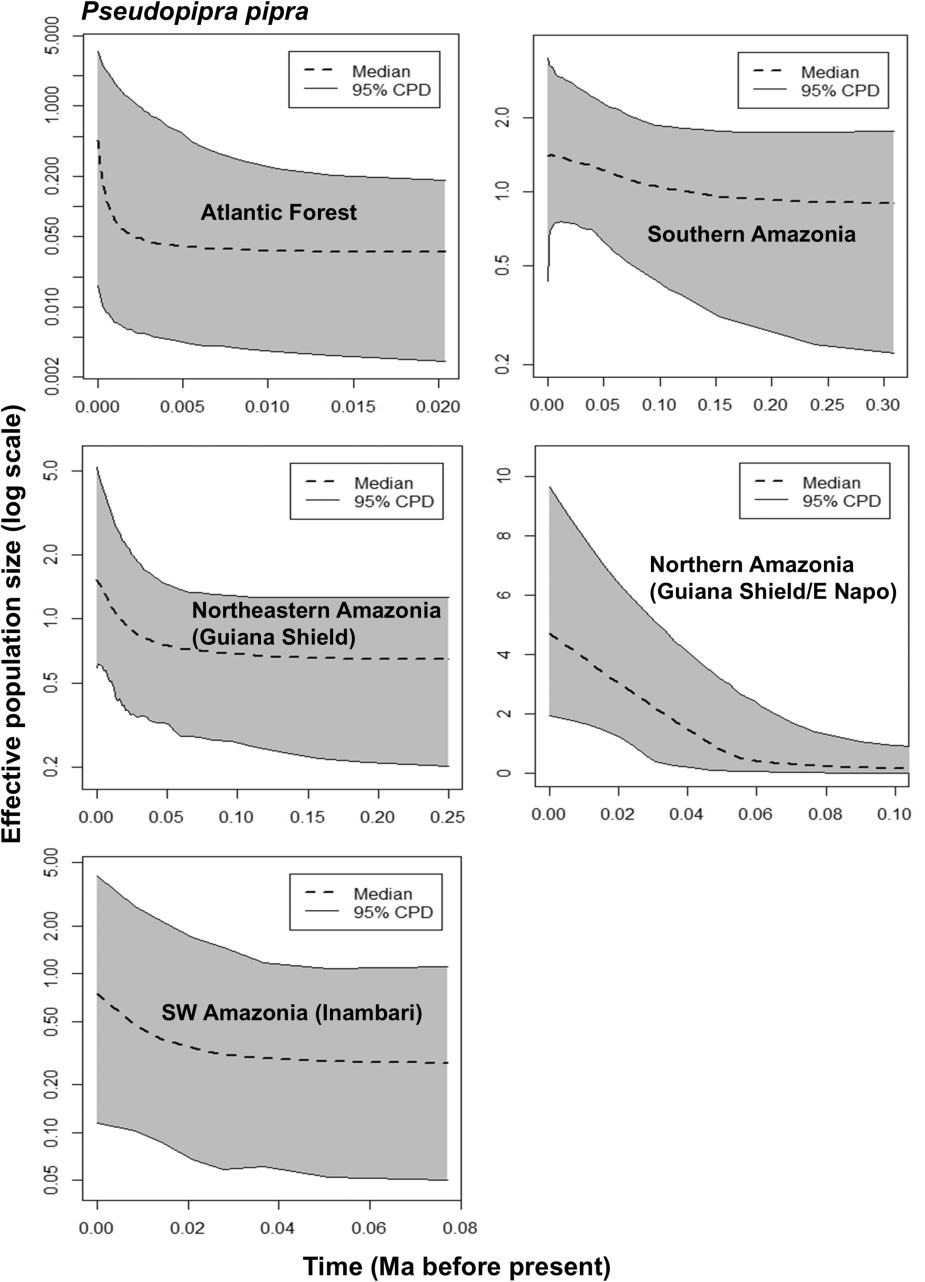
Demographic history of *Pseudopipra pipra* lineages inferred through extended Bayesian skyline plots (EBSPs) estimated in BEAST, based on ND2 sequence data. Dashed lines depict median population size, area in gray denotes 95% Bayesian Credible Intervals. Time scale in millions of years before present.

For both species, the EBSP analyses indicated a trend of increasing population sizes over time in the Atlantic Forest (Figures 4 and 5). For *C. rubrocapilla*, the EBSP suggests increased population growth starting approximately 10,000 years before present (Figure 4), whereas for *P. pipra* the increase is estimated to have happened more recently, ca. 2500 years before present (Figure 5). The Atlantic Forest populations of both *C. rubrocapilla* and *P. pipra* have small positive values of Tajima's *D*, Fu's *F*s, and Ramos‐Onsins & Rozas *R*2 statistics, which are not expected under a scenario of population size expansion, but none were significant (Table 1).

In contrast to the Atlantic Forest lineages, results from both species suggest a more gradual population size increase through time in the Amazon region (Figures 4 and 5). The Amazonian population of *C. rubrocapilla* and the Amazonian populations of *P. pipra* , (with the exception of southwestern Amazonia) have negative values of Tajima's *D* statistic and Fu's *F*s statistics and small values of the Ramos‐Onsins & Rozas *R*2 statistic, which indicate gradual population increases, although not all were significant (Table 1). The southwestern Amazonian population of *P. pipra* has positive Tajima's *D* and low Fu's *F*s and Ramos‐Onsins & Rozas *R*2 statistics, but those numbers were not statistically significant (Table 1).

### 3.4 Climatic Modeling

**FIGURE 6:**
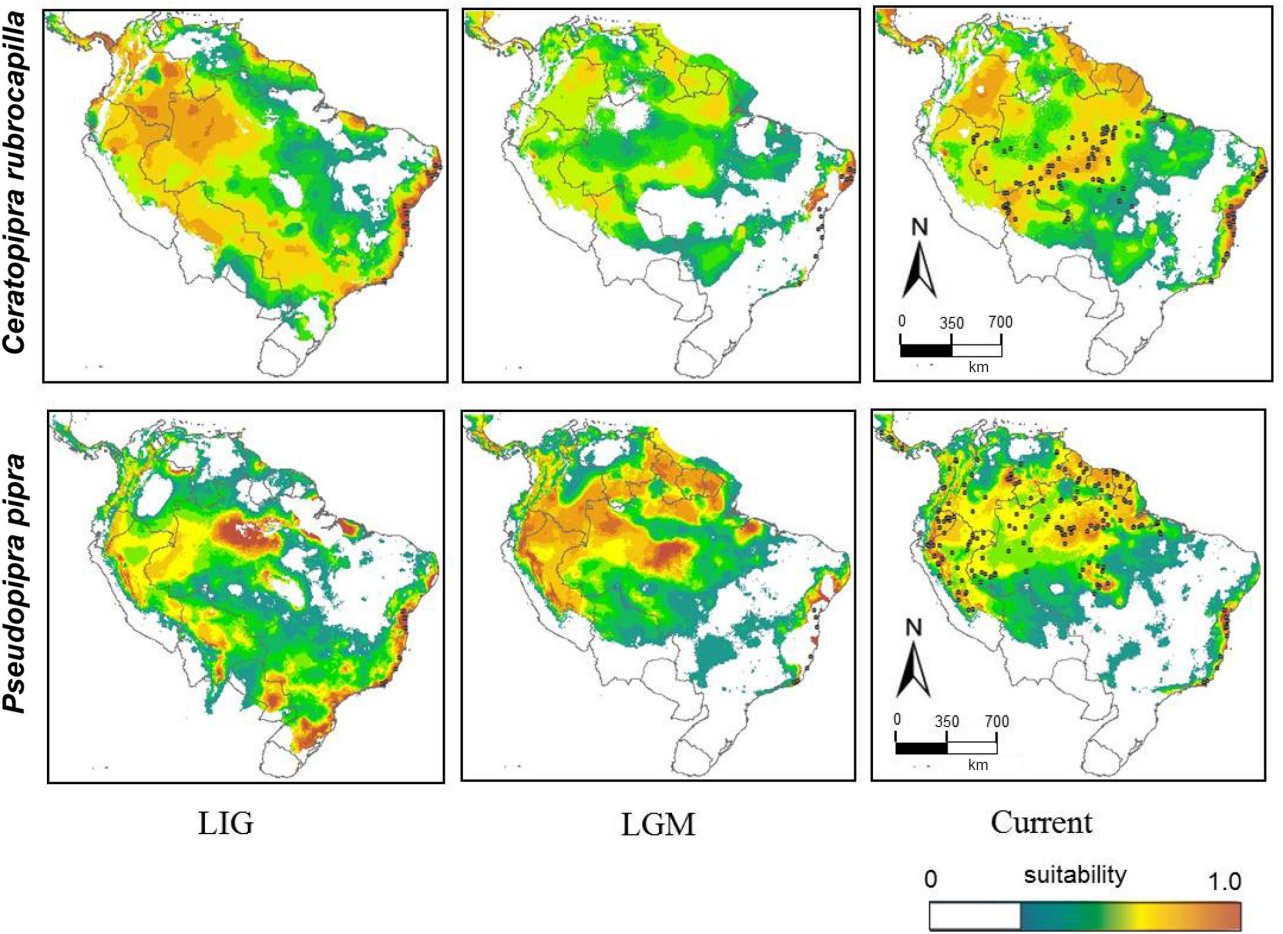
Modeled suitable climatic conditions for *Ceratopipra rubrocapilla* and *Pseudopipra pipra* across Quaternary climatic fluctuations, and current climate. Green color indicates low predicted suitability, yellow to red colors indicate higher values, and white areas indicate those pixels with values below the minimum training presence (MTP) threshold, as determined based on the calibration data. Dots on the current climate map depict localities of known species occurrence; dots on the Atlantic Forest region indicate localities for which genetic data were collected (LGM, last glacial maximum; LIG, last interglacial maximum).

For *C. rubrocapilla*, the best model parameter set with low test‐omission rate and high test‐AUC consisted of a hinge (*H*) feature class and a regularization value of 3.5. For *P. pipra* , the best models were based on three feature classes (linear‐quadratic‐hinge, or LQH) and regularization multiplier 1. SDMs developed under current climatic conditions showed good performance for both species, especially for *P. pipra* . Regarding the threshold‐independent measures, the highest AUC evaluation (highest overall performance) was 0.791 for *C. rubrocapilla* and 0.987 for *P. pipra* . For the threshold‐dependent measures, the lowest omission rate (lowest overfitting leading to optimal complexity) was 0.196 for *C. rubrocapilla* and 0.298 for *P. pipra* . The models nonetheless predicted *C. rubrocapilla* to occur in areas where it is not currently found (Figure 6). This includes the northern region of the Amazonian forests (Southeastern Ecuador, Northeastern Peru, Colombia, Southwestern Venezuela, French Guiana, Suriname, and Guyana), and even the regions west of the Andes in Southwestern Ecuador, as well as in Panamá and Costa Rica.

For *C. rubrocapilla*, projections onto the LGM suggest that suitable conditions were more continuously distributed throughout Amazonia (except in the central and in the northern part of Brazil) and infer more fragmentation throughout the Atlantic coast. For *P. pipra* , inferred LGM suitability in the Amazon was similar to today's, whereas a scenario of fragmentation is inferred throughout the Atlantic coast. Projections onto the LIG support a potential range for *C. rubrocapilla* that is similar to today's, and a slight contraction in Amazonia for *P. pipra* .

The paleoclimatic models also provided some insight about the potential for connectivity between Amazonian and Atlantic Forest manakins. For *C. rubrocapilla*, the LIG model indicates the existence of areas with high suitability connecting the Southern Amazon with the southern Atlantic forests, through a corridor that crosses the interior states of Mato Grosso, Mato Grosso do Sul, and Goiás (Figure 6). For *P. pipra* , the model indicates a similar and broad potential connection during the LIG, with low to moderate suitability values in areas between the southwestern Amazon and the southern Atlantic Forest (Figure 6). A connection between eastern Amazonia and the northeastern region of the Atlantic Forest was not recovered in any time period or climatic reconstruction examined.

## 4 Discussion

Studies of historical demography and phylogenetic reconstruction that rely on DNA sequence information from a single gene, although accessible and relatively inexpensive, are known to portray a partial view of the processes impacting the genome of the species of study, and to result in inferences with wide confidence intervals (Galtier et al. 2009). We acknowledge this limitation and, to circumvent these challenges, contrast our results with descriptions of the local landscape, the taxonomy, other studies based on more genetic loci, and distribution models of the two species of manakins studied here, using congruency across datasets as validation. For instance, we found little mtDNA structure within the Amazonian clade of *C. rubrocapilla*, whose haplotypes are not differentiated across major rivers or centers of endemism. This finding is congruent with the lack of recognized subspecies in this taxon and the results obtained by Mikkelsen et al. (2024). In contrast, we found high mtDNA structure within *P. pipra* , particularly in Amazonia, where several major lineages are bounded by rivers (Figure 2), and where local subspecies have been traditionally recognized. Still, the phylogenetic placement of several individual samples suggests the occurrence of introgression (e.g., in clades VI, VII, and X of Figure 2), which agrees with Berv et al.'s (2021) phylogenomic study.

### 4.1 Amazonia and Atlantic Forest Relationships

In *C. rubrocapilla*, our mitochondrial analyses show that Amazonian populations are closely related to those of the Atlantic Forest (Figure 1). Although support for the Amazonian and Atlantic Forest clades was low, the divergence time estimate for the Amazonian‐Atlantic Forest split is recent, which could explain the lack of support by having not enough time for lineage sorting (see below). mtDNA analyses of *P. pipra* found that the Atlantic Forest clade is embedded within a large Amazonian clade (Figures 2 and 3) that included six Amazonian lineages. Phylogenetic relationships of the Atlantic Forest lineage to the Amazonian ones were not resolved by the mtDNA tree. However, Berv et al. (2021) ddRAD phylogenomic study of *P. pipra* found strong support for the Atlantic Forest being close to SE Amazonia. A similar pattern has been documented in other avian species and closely related taxa (Cracraft and Prum 1988; Bates, Hackett, and Cracraft 1998; Marks, Hackett, and Capparella 2002; Ribas and Miyaki 2004; Nyári 2007; Prates et al. 2016; Dantas et al. 2021; Simões et al. 2022).

The mtDNA tree and the diversity levels of *P. pipra* populations support the hypothesis that the species originated in Amazonia and dispersed to the Atlantic Forest (Figures 2 and 3). Although the time of the origin of the Atlantic Forest clade was not resolved, is expected to have occurred in the Early to Middle Pleistocene (in between nodes 8 and 9 of Figure 3). The Atlantic Forest lineage has lower overall nucleotide diversity and shallower geographic structure relative to the Amazonian *P. pipra* haplogroups, in agreement with a more recent origin. For *C. rubrocapilla*, the split between Amazonia and the Atlantic Forest is also dated at the Middle Pleistocene (0.4 [0.5–0.2] Ma), overlapping with the estimates of Mikkelsen et al. (2024) for the separation between haplotypes 1 (Atlantic Forest + eastern Amazonia) and 2 (Amazonia) in their cytochrome *b* tree (0.34–0.71 Ma), and at a similar time of the inferred Atlantic Forest colonization by *P. pipra* (in between nodes 8 and 9 of Figure 3 [0.9–0.2 Ma]).

Recent phylogenetic analyses of passerine birds have suggested that faunal interchange happened through similarly young connections between the Amazon and the Atlantic Forest, either through regions presently characterized by drier Cerrado and Caatinga habitats, or via the coast of northeastern Brazil (Batalha‐Filho, Fjeldså, et al. 2013; Rocha et al. 2015; Thom and Aleixo 2015; Dantas et al. 2021; Simões et al. 2022). Although the molecular data collected here are consistent with the existence of such a recent connection, the lack of documented populations (and hence samples) from forest enclaves and gallery forests within the Caatinga and Cerrado precludes us from distinguishing between these two possible pathways. One Amazonian sample of *C. rubrocapilla* (from Pará) was embedded with the Atlantic Forest clade (Figure 1a,b), and also inferred to belong in the Atlantic Forest haplogroup (Figure 1c). This pattern was also observed extensively in the mitochondrial dataset of Mikkelsen et al. (2024), reinforcing the recency of the connection between these two biomes.

### 4.2 Integrating Historical Population Demography and Climatic Modeling

The SDMs developed here provide insights into the drivers of present‐day range size in the study taxa (Figure 6). The model of *C. rubrocapilla*, for instance, identifies suitable areas that are not presently occupied by the species. These include areas in northwestern Bolivia and southeastern Peru, where the sister taxon, Round‐tailed Manakin (*C. chloromeros*), is found, and also north of the Amazon River, an area that is occupied by a closely related species *C. erythrocephala* . We hypothesize that these portions of the environment are not being occupied by the species due to biotic interactions at play, and not due to climatic constraints: the fact that *C. rubrocapilla* does not occur north of the Amazon River may be explained by the local presence of a related species, *C. erythrocephala* . This is supported by some indication of potential admixture between *Ceratopipra* species inferred from genomic data (Harvey et al. 2017). In *P. pipra* , however, this is not observed. The present‐day environmental model for *P. pipra* is well matched to the actual geographic distribution of the species.

The models also provide insights about potential connections between forest domains, and the potential role of Late Quaternary climate change on the distribution of genetic diversity within the study taxa (Figure 6). For instance, none of the models projected to the past (21, 120 ka) recovered a connection between eastern Amazonia and the northeastern region of the Atlantic Forest, as suggested by Batalha‐Filho, Fjeldså, et al. (2013). Instead, distribution models applied to the LIG period, in both species, detected an area of suitability connecting southwestern Amazonia to the southern Atlantic Forest (Figure 6), which is similar to Cabanne et al.'s (2019) SDM for *Syndactyla rufosuperciliata* that suggested a contact between the Andean and the Atlantic Forest through the Cerrado during peaks of glacial periods. The colonization routes suggested for our study taxa and *S. rufosuperciliata* are similar to one of the connections inferred by Batalha‐Filho, Fjeldså, et al. (2013) yet differ in its inferred timing; Batalha‐Filho, Fjeldså, et al. (2013) recovered southern connections in earlier time periods (Late to Middle Miocene), which raises the possibility that these connections may have happened at multiple times, including the Late Pleistocene, through events that may have played an important role in the evolutionary history of forest organisms within the South American dry diagonal (Ledo et al. 2020). That being said, we did not find concordance between the timing and placement of the potential forest connection inferred through the paleoclimatic model (SW Amazonia—S Atlantic Forest, 120 ka) and the cross‐forest colonization inferred by the molecular data (e.g., Amazonia to Atlantic Forest colonization between 0.9 to 0.2 Ma in *P. pipra* ).

The LGM model projections for both species suggest more environmental suitability in Amazonia when compared to the Atlantic Forest, and compared to present‐day distributions, indicating a recent (post‐LGM) southward expansion of suitable areas, especially in the Atlantic Forest. This scenario is consistent with the results of the population demography analysis, which, despite having wide 95% conditional probability distributions, suggests demographic stability in Amazonia and signatures of population size change (expansion) within the Atlantic Forest for both species, (during the last 10,000 years for *C. rubrocapilla* and 2500 years for *P. pipra* ; Figures 4 and 5). This is also consistent with the observations that nucleotide diversity is higher in Amazonian populations when compared to those in the Atlantic Forest, consistent with the findings of Mikkelsen et al. (2024).

Collectively, our results demonstrate that no simple hypothesis explains biodiversity patterns in Neotropical wet forests (see Bush 1994; Haffer 1997; Dantas, Cabanne, and Santos 2011). The genetic data and SDMs of *C. rubrocapilla* and *P. pipra* show that phylogeographic patterns are congruent between these two species in just a subset of their ranges: the Atlantic Forest. Levels and patterns of genetic and taxonomic structure in Amazonia differ widely between the taxa: older *P. pipra* has a more marked genetic structure and taxonomic differentiation relative to the younger *C. rubrocapilla*. Paleoclimatic models going back to the LIG identify a potential SW Amazonia—S Atlantic Forest connection in both taxa, but no pathways through the north. Genealogical analyses nonetheless infer colonization from Amazonia to the Atlantic Forest in *P. pipra* , dating to 0.7 Ma (Figure 3), which is also supported for *C. rubrocapilla* based on a different dataset (Mikkelsen et al. 2024). This suggests that multiple corridors, and at different locations, may have been available to Neotropical bird species over the Pliocene and Pleistocene—although we have yet to understand why some of them resulted in gene flow, whereas others may not (see Mikkelsen et al. 2024). The establishment and use of forest corridors by bird species may have been more complex relative to the dynamics described in the literature (e.g., Batalha‐Filho, Irestedt, et al. 2013). Further studies, including more samples and genomic‐scale sampling, are needed to understand differences in the dynamics and historical demography of Amazonian and Atlantic Forest populations.

## Author Contributions

**Ivandy N. Castro‐Astor:** conceptualization (lead), writing – original draft (lead). **Ana Carolina Carnaval:** supervision (lead), writing – review and editing (supporting). **José G. Tello:** supervision (supporting), writing – review and editing (supporting). **Maria Alice S. Alves:** resources (supporting), writing – review and editing (supporting). **Joel Cracraft:** resources (supporting), supervision (lead). **Alexandre Aleixo:** resources (supporting). **William M. Mauck III:** resources (supporting). **Charles Duca:** resources (supporting).

## Conflicts of Interest

The authors declare no conflicts of interest.

## Supporting information


Data S1.

## Data Availability

The sequence data that support the findings of this study are available in GenBank. Accession numbers are provided in Supporting Information. [We are submitting the data matrices to Dryad Digital Repository, and we will include the link to the site shortly. Unfortunately, Dryad Digital Repository requires a manuscript number before processing a dataset submission.]
