# Supplementary material for: Phylogeography, Historical Population Demography, and Climatic Modeling of Two Bird Species Uncover Past Connections Between Amazonia and the Atlantic Forest
Source: Ecol Evol. 2024 Dec 9;14(12):e70587. doi: 10.1002/ece3.70587 (PMC11628634; doi:10.1002/ece3.70587)
Supplement: Supplementary file 1 — Data S1. [file ECE3-14-e70587-s001.docx]

**Supporting Information**

**For**

**Phylogeography, historical population demography, and climatic modelling of two forest birds uncover past connections between Amazonia and the Atlantic Forest**

Ivandy N. Castro-Astor^1^, Joel Cracraft^2^, José G. Tello^2,3^, Maria Alice S. Alves^4^, William M. Mauck III^2^, Alexandre Aleixo^5^, Charles Duca^6^, Ana Carolina Carnaval^1,7^

^1^Department of Biology, City College of New York and CUNY Graduate Center, City University of New York, New York, NY 10031, USA

^2^Department of Ornithology, American Museum of Natural History, Central Park West at 79th St., New York, NY 10024, USA

^3^Department of Biology, Long Island University, 1 University Plaza, Brooklyn, NY 11201, USA

4Departamento de Ecologia, Universidade do Estado do Rio de Janeiro, Rua São Francisco Xavier, 524 - PHLC, sala 224, Maracanã, Rio de Janeiro, RJ 20.550-013, Brazil

^5^Coordenação de Zoologia, Museu Paraense Emílio Goeldi, Av. Magalhães Barata, 376, São Braz, Belém, PA 66.040-170, Brasil

^6^Universidade Vila Velha, Unidade Acadêmica II - Biomédicas, Boa Vista II, Vila Velha, ES, 29.1029-20, Brazil

^7^CUNY Graduate Center, 365 Fifth Avenue, New York, NY 10016, USA

*Corresponding author. E-mail: ivandyastor@gmail.com

**Table S1.** Sampling localities of *Ceratopipra rubrocapilla* and *Pseudopipra pipra*, including geographic coordinates, sample size per locality, and voucher numbers**.**

| Species | Locality | Latitude | Longitude | Voucher | GenBank |
| --- | --- | --- | --- | --- | --- |
| *Ceratopipra rubrocapilla* | Reserva Biológica União, Rio de Janeiro State, Brazil | -22.4167 | -42.0333 | UERJ C24526^b^ | PQ272337 |
|  | Reserva Biológica União, Rio de Janeiro State, Brazil | -22.4167 | -42.0333 | UERJ C24536^b^ | PQ272338 |
|  | Reserva Biológica União, Rio de Janeiro State, Brazil | -22.4167 | -42.0333 | UERJ C24547^b^ | PQ272339 |
|  | Reserva Biológica União, Rio de Janeiro State, Brazil | -22.4167 | -42.0333 | UERJ C24782^b^ | PQ272340 |
|  | Reserva Biológica União, Rio de Janeiro State, Brazil | -22.4167 | -42.0333 | UERJ D51919^b^ | PQ272342 |
|  | Fazenda Cupido e Refúgio, Linhares, Espírito Santo State, Brazil | -19.053611 | -39.971028 | MZFS100003^b^ | PQ272343 |
|  | Ilhéus, Ecoparque de UNA, Bahia State, Brazil | -15.159722 | -39.045 | MPEG 70775^l^ | PQ272359 |
|  | Ilhéus, Ecoparque de UNA, Bahia State, Brazil | -15.159722 | -39.045 | MPEG 70789^l^ | PQ272362 |
|  | Ilhéus, Ecoparque de UNA, Bahia State, Brazil | -15.159722 | -39.045 | MPEG 70776^l^ | PQ272360 |
|  | Ilhéus, Ecoparque de UNA, Bahia State, Brazil | -15.159722 | -39.045 | MPEG 70777^l^ | PQ272361 |
|  | Ilhéus, Ecoparque de UNA, Bahia State, Brazil | -15.159722 | -39.045 | MPEG 70778^l^ | PQ272344 |
|  | Ilhéus, Ecoparque de UNA, Bahia State, Brazil | -15.159722 | -39.045 | MPEG 70790^l^ | PQ272363 |
|  | Restinga Trancoso, Bahia State, Brazil | -16.5333 | -39.1 | UERJ D38947^b^ | PQ272341 |
|  | Igrapiúna, Reserva da Michelin, Bahia State, Brazil | -13.8333 | -39.2333 | MZFS 00438^b^ | PQ272348 |
|  | Igrapiúna, Reserva da Michelin, Bahia State, Brazil | -13.8333 | -39.2333 | MZFS 00599^b^ | PQ272349 |
|  | Igrapiúna, Reserva da Michelin, Bahia State, Brazil | -13.8333 | -39.2333 | MZFS 00651^b^ | PQ272350 |
|  | Igrapiúna, Reserva da Michelin, Bahia State, Brazil | -13.8333 | -39.2333 | MZFS 00652^b^ | PQ272351 |
|  | Santa Terezinha, Serra da Jiboia, Bahia State, Brazil | -12.85 | -39.4667 | MZFS 739^b^ | PQ272352 |
|  | Santa Terezinha, Serra da Jiboia, Bahia State, Brazil | -12.85 | -39.4667 | MZFS 740^b^ | PQ272353 |
|  | Santa Terezinha, Serra da Jiboia, Bahia State, Brazil | -12.85 | -39.4667 | MZFS 741^b^ | PQ272354 |
|  | Santa Terezinha, Serra da Jiboia, Bahia State, Brazil | -12.85 | -39.4667 | MZFS 742^b^ | PQ272355 |
|  | Santa Terezinha, Serra da Jiboia, Bahia State, Brazil | -12.85 | -39.4667 | MZFS 744^b^ | PQ272356 |
|  | Santa Terezinha, Serra da Jiboia, Bahia State, Brazil | -12.85 | -39.4667 | MZFS 749^b^ | PQ272357 |
|  | Santa Terezinha, Serra da Jiboia, Bahia State, Brazil | -12.85 | -39.4667 | MZFS 753^b^ | PQ272358 |
|  | RPPN Serra Bonita, Fazenda Paris, Camacan, Bahia State, Brazil | -15.4167 | -39.5333 | MZUSP 91029^l^ | PQ272345 |
|  | RPPN Serra Bonita, Fazenda Paris, Camacan, Bahia State, Brazil | -15.4167 | -39.5333 | MZUSP 91030^l^ | PQ272364 |
|  | RPPN Serra Bonita, Fazenda Paris, Camacan, Bahia State, Brazil | -15.4167 | -39.5333 | MZUSP 91031^l^ | PQ272346 |
|  | Timbauba, Pernambuco State, Brazil, Brazil | -7.6 | -35.3667 | FMNH 392359l | PQ272306 |
|  | Timbauba, Pernambuco State, Brazil, Brazil | -7.6 | -35.3667 | FMNH 392360^l^ | PQ272336 |
|  | Timbauba, Pernambuco State, Brazil, Brazil | -7.6 | -35.3667 | FMNH 392361^l^ | PQ272309 |
|  | Timbauba, Pernambuco State, Brazil, Brazil | -7.6 | -35.3667 | FMNH 392362^l^ | PQ272310 |
|  | Timbauba, Pernambuco State, Brazil, Brazil | -7.6 | -35.3667 | FMNH 392363^l^ | PQ272311 |
|  | Timbauba, Pernambuco State, Brazil, Brazil | -7.6 | -35.3667 | FMNH 392364^l^ | PQ272312 |
|  | Serra do Espelho, Pernambuco State, Brazil | -7.81667 | -34.85 | FMNH 392452^l^ | PQ272313 |
|  | Serra do Espelho, Pernambuco State, Brazil | -7.81667 | -34.85 | FMNH 392453^l^ | PQ272314 |
|  | Mata do Estado, Pernambuco State, Brazil | -7.58333 | -35.5 | FMNH 427165^l^ | PQ272315 |
|  | Mata do Estado, Pernambuco State, Brazil | -7.58333 | -35.5 | FMNH 427166^l^ | PQ272316 |
|  | Mata do Estado, Pernambuco State, Brazil | -7.58333 | -35.5 | FMNH 427167^l^ | PQ272317 |
|  | Mata do Estado, Pernambuco State, Brazil | -7.58333 | -35.5 | FMNH 427168^l^ | PQ272318 |
|  | Mata do Estado, Pernambuco State, Brazil | -7.58333 | -35.5 | FMNH 427169^l^ | PQ272319 |
|  | Mata do Estado, Pernambuco State, Brazil | -7.58333 | -35.5 | FMNH 427170^l^ | PQ272320 |
|  | Mata do Estado, Pernambuco State, Brazil | -7.58333 | -35.5 | FMNH 427171^l^ | PQ272321 |
|  | Mata do Estado, Pernambuco State, Brazil | -7.58333 | -35.5 | FMNH 427172^l^ | PQ272322 |
|  | Mata do Estado, Pernambuco State, Brazil | -7.58333 | -35.5 | FMNH 427173^l^ | PQ272323 |
|  | Macambira, Pernambuco State, Brazil | -8.6 | -36.4333 | FMNH 427174^l^ | PQ272324 |
|  | Barreiros, Engenho Cachoeira Linda, Pernambuco State, Brazil | -8.81667 | -35.4667 | MPEG 72131^1^ | PQ272335 |
|  | Ibateguara, Engenho Coimbra, Usina Serra Grande, Alagoas State, Brazil | -8.96667 | -35.85 | FMNH 427175^l^ | PQ272325 |
|  | Ibateguara, Engenho Coimbra, Usina Serra Grande, Alagoas State, Brazil | -8.96667 | -35.85 | F FMNH 427176^l^ | PQ272326 |
|  | Ibateguara, Engenho Coimbra, Usina Serra Grande, Alagoas State, Brazil | -8.96667 | -35.85 | FMNH 427177^l^ | PQ272327 |
|  | Ibateguara, Engenho Coimbra, Usina Serra Grande, Alagoas State, Brazil | -8.96667 | -35.85 | FMNH 427178^l^ | PQ272328 |
|  | Ibateguara, Engenho Coimbra, Usina Serra Grande, Alagoas State, Brazil | -8.96667 | -35.85 | FMNH 427179^l^ | PQ272307 |
|  | Ibateguara, Engenho Coimbra, Usina Serra Grande, Alagoas State, Brazil | -8.96667 | -35.85 | FMNH 427180^l^ | PQ272329 |
|  | Ibateguara, Engenho Coimbra, Usina Serra Grande, Alagoas State, Brazil | -8.96667 | -35.85 | MPEG 70502^l^ | PQ272347 |
|  | Ibateguara, Engenho Coimbra, Usina Serra Grande, Alagoas State, Brazil | -8.96667 | -35.85 | MPEG 70500^l^ | PQ272330 |
|  | Ibateguara, Engenho Coimbra, Usina Serra Grande, Alagoas State, Brazil | -8.96667 | -35.85 | MPEG 70504^l^ | PQ272331 |
|  | Ibateguara, Engenho Coimbra, Usina Serra Grande, Alagoas State, Brazil | -8.96667 | -35.85 | MPEG 70503 ^l^ | PQ272365 |
|  | Ibateguara, Engenho Coimbra, Usina Serra Grande, Alagoas State, Brazil | -8.96667 | -35.85 | MPEG 70501^l^ | PQ272308 |
|  | Ibateguara, Engenho Coimbra, Usina Serra Grande, Alagoas State, Brazil | -8.96667 | -35.85 | MPEG 70505^l^ | PQ272332 |
|  | Ibateguara, Engenho Coimbra, Usina Serra Grande, Alagoas State, Brazil | -8.96667 | -35.85 | MPEG 70506^l^ | PQ272333 |
|  | Ibateguara, Engenho Coimbra, Usina Serra Grande, Alagoas State, Brazil | -8.96667 | -35.85 | MPEG 70507^l^ | PQ272334 |
|  | Canarana, Fazenda Tanguro, Mato Grosso State, Brazil | -12.8833 | -52.3667 | MPEG 60340^l^ | PQ272366 |
|  | Canarana, Fazenda Tanguro, Mato Grosso State, Brazil | -12.8833 | -52.3667 | MPEG 60341^l^ | PQ272367 |
|  | Novo Progresso, Base Aeronaútica Serra do Cachimbo, Torre II, Pará State, Brazil | -9.2667 | -54.9333 | MPEG 57985^1^ | PQ272379 |
|  | Parque Estadual Sucunduri, margem direita do rio Bararati, Amazonas State, Brazil | -8.35 | 58.61666667 | INPAA 0770^l^ | PQ272368 |
|  | Manicoré, Rodovia do Estanho, km 136, Amazonas State, Brazil | -8.68333 | -61.4 | MPEG 57761^l^ | PQ272369 |
|  | Belterra, Flona do Tapajós, Santarém/Cuiabá, BR 163 Km 117, Pará State, Brazil | -3.35 | -54.9333 | MPEG 56127^l^ | PQ272370 |
|  | ca 45 km sudoeste de Porto Velho, margem esquerda do Rio Madeira, Rondonia State Brazil | - 9.177778 | - 64.383333 | INPAA 0341^l^ | PQ272371 |
|  | Porto Walter, Igarapé Cruzeiro do Vale, Colônia Dois Portos, Acre State, Brazil | -8.33333 | -72.6 | MPEG 62160^l^ | PQ272373 |
|  | Rio Ouro Preto, margem esquerda, Guajará-Mirim, Reserva Biológica Ouro Preto, Rondonia State, Brazil | -10.8333 | -64.75 | MPEG 55065^l^ | PQ272374 |
|  | Tefé, Base Petrobras/Urucu, Igarapé Lontra, Amazonas State, Brazil | -5.9333 | -71.5333 | MPEG 57187^l^ | PQ272375 |
|  | RDS, Cujubim, ca 390 km SW Jataí, Amazonas State, Brazil | -5.21667 | -68.3167 | MPEG 60256^l^ | PQ272376 |
|  | Itaituba, FLONA Amanã, Garimpo JMS, margem direita Igarapé Porquinho, Pará State | -5.1 | -57.5333 | MPEG 65153^l^ | PQ272377 |
|  | Jacareacanga, Transamazônica, ponte sobre o Rio Igarapé Preto, Pará State | -5.9 | -57.6833 | MPEG 65645^l^ | PQ272378 |
|  | Campo do Lago Preto, margem esquerda do Rio Madeira, 39 km W Novo Aripuanã, Amazonas State, Brazil | -5.161111 | -60.744444 | INPAA 0418^b^ | PQ272372 |
|  | Floresta Nacional de Caxiuanã, Melgaço, Pará, Brazil | -1.708333 | -51.529167 | - | KF228555 |
| *Pseudopipra pipra* | Reserva Biológica União, Rio de Janeiro State, Brazil, Brazil | -22.4167 | -42.0333 | - | MW771050 |
|  | Reserva Biológica União, Rio de Janeiro State, Brazil, Brazil | -22.4167 | -42.0333 | UERJ C23549^b^ | PQ272289 |
|  | Reserva Biológica União, Rio de Janeiro State, Brazil, Brazil | -22.4167 | -42.0333 | UERJ C23594^b^ | PQ272290 |
|  | Reserva Biológica União, Rio de Janeiro State, Brazil, Brazil | -22.4167 | -42.0333 | - | MW771051 |
|  | Reserva Biológica União, Rio de Janeiro State, Brazil, Brazil | -22.4167 | -42.0333 | UERJ C24516^b^ | PQ272291 |
|  | Reserva Biológica União, Rio de Janeiro State, Brazil, Brazil | -22.4167 | -42.0333 | UERJ C24519^b^ | PQ272292 |
|  | Reserva Biológica União, Rio de Janeiro State, Brazil, Brazil | -22.4167 | -42.0333 | UERJ C24520^b^ | PQ272293 |
|  | Reserva Biológica União, Rio de Janeiro State, Brazil, Brazil | -22.4167 | -42.0333 | UERJ C24521^b^ | PQ272294 |
|  | Reserva Biológica União, Rio de Janeiro State, Brazil, Brazil | -22.4167 | -42.0333 | UERJ C24531^b^ | PQ272295 |
|  | Reserva Biológica União, Rio de Janeiro State, Brazil, Brazil | -22.4167 | -42.0333 | UERJ C24534^b^ | PQ272296 |
|  | Reserva Biológica União, Rio de Janeiro State, Brazil, Brazil | -22.4167 | -42.0333 | UERJ C24541^b^ | PQ272297 |
|  | Reserva Biológica União, Rio de Janeiro State, Brazil, Brazil | -22.4167 | -42.0333 | UERJ C24546^b^ | PQ272298 |
|  | Reserva Biológica União, Rio de Janeiro State, Brazil, Brazil | -22.4167 | -42.0333 | UERJ C24554^b^ | PQ272299 |
|  | Reserva Biológica União, Rio de Janeiro State, Brazil, Brazil | -22.4167 | -42.0333 | UERJ D30786^b^ | PQ272301 |
|  | Reserva Biológica União, Rio de Janeiro State, Brazil, Brazil | -22.4167 | -42.0333 | UERJ D30790^b^ | PQ272302 |
|  | Reserva Biológica União, Rio de Janeiro State, Brazil, Brazil | -22.4167 | -42.0333 | UERJ D30794^b^ | PQ272303 |
|  | Reserva Biológica União, Rio de Janeiro State, Brazil, Brazil | -22.4167 | -42.0333 | UERJ D51928^b^ | PQ272304 |
|  | Reserva Biológica União, Rio de Janeiro State, Brazil, Brazil | -22.4167 | -42.0333 | - | MW771054 |
|  |  |  |  | UERJ D51931^b^ | PQ272305 |
|  | Reserva Biológica União, Rio de Janeiro State, Brazil, Brazil | -22.4167 | -42.0333 | - | MW771052 |
|  | Reserva Biológica de Poço das Antas, Rio de Janeiro State, Brazil | -22.3 | -42.1 | UERJ D30777^b^ | PQ272300 |
|  | Fazenda Cupido e Refúgio-Linhares, Espírito Santo State, Brazil | -19.053611 | -39.971028 |  | MW771023 |
|  | Santa Terezinha, Serra da Jiboia, Bahia State, Brazil, Brazil, Brazil | -12.85 | -39.4667 | DA 00653 | PQ272267 |
|  | Margem esquerda do Rio Negro, ca 10 km E São Gabriel da Cachoeira, estrada da Olaria, Amazonas State, Brazil | -0.12000000 | -67.08194444 | INPAA 1121^l^ | PQ272276 |
|  | Rio Araca, Barcelos, Amazonas State, Brazil | -0.4167 | -60.9333 | AMNH DOT14580^l^ | PQ272275 |
|  | Parque Nacional do Jaú; margem esquerda Rio Jaú, comunidade "Seringalzinho", base Tiaracá, Amazonas State, Brazil | -1.8835 | -61.7375 | INPA A1965^l^ | PQ272279 |
|  | Parque Nacional Viruá, "Sede", 35 km SSE Caracaraí, Roraima State, Brazil | -1.81611111 | -61.12805556 | INPAA 1085^l^ | PQ272278 |
|  | Óbidos, Flota do Trombetas, Pará State, Brazil | -0.95 | -55.5167 | MPEG 65071^l^ | PQ272280 |
|  | ESEC Juami-Japurá; margem direita do Rio Japurá; baixo Rio Juami, ca 94 km W Japurá, Amazonas State, Brazil | -1.65555556 | -68.06388889 | INPAA 0693^l^ | PQ272277 |
|  | Margem direita do Rio Demini, "Demini Camp", ca 105 km N Barcelos, Amazonas State, Brazil | -0.7833 | -63.15 | INPAA 1255^l^ | PQ272281 |
|  | 15 km WSW Caracarai; margem esquerda R Branco, vicinal Agua Boa, Rondonia State, Brazil | -1.70694444 | -61.17333333 | INPAA 1682^l^ | PQ272282 |
|  | 110 km ENE Santa Isabel do Rio Negro; margem esquerda do Rio Preto, "Comunidade campina do Rio Preto, castanhal", Amazonas State, Brazil | -0.4 | -65.03333333 | INPAA 1601^l^ | PQ272283 |
|  | Município de Santa Bárbara, GUNMA, Pará State, Brazil | -1.1833 | -48.2833 | MPEG 59100^l^ | PQ272286 |
|  | Marajó, Breves, Sítio do Waldir | -1.55 | -50.3833 | MPEG 61143^l^ | PQ272287 |
|  | Portel, FLONA do Caxiuanã, Plot PPBIO, Pará State, Brazil | -1.95 | -51.6 | MPEG 61918^l^ | PQ272288 |
|  | Novo Progresso, Base Aeronaútica Serra do Cachimbo, Torre II, Pará State, Brazil | -9.26667 | -54.9333 | MPEG 57978^l^ | PQ272285 |
|  | Parque Estadual Sucunduri; margem direita R. Bararati, Amazonas , Brazil | -8.35 | -58.61666667 | INPA A0858^l^ | PQ272284 |
|  | Feijó, Rio Envira, Novo Porto, Foz do Ig. Paraná do Ouro, Acre State, Brazil | -8.45 | -70.55 | MPEG 63810^l^ | PQ272271 |
|  | Tarauacá, Floresta Estadual Rio Gregório, próximo ponte Rio Acuraua, Acre State, Brazil | -8.067944 | -71.176944 | MPEG 60800^1^ | PQ272269 |
|  | Alenquer, ESEC Grão, Pará State, Brazil | -0.15 | -55.18333333 | MPEG 65492 | PQ272273 |
|  | Estrada Manacapuru-Novo Airão Km75, Amazonas State, Brazil | -2.85 | -60.85 | AMNH DOT14157 | PQ272274 |
|  | União Biological Reserve, Rio de Janeiro, Brazil | -22.4167 | -42.0333 | D 51931 | PQ272305 |
|  | Resex Baixo Juruá, Amazonas State, Brazil | -3.72777778 | -66.09444444 | INPA A0812 | PQ272268 |
|  | Tarauacá, Floresta Estadual do Mogno, Br 364 km 6 Rio Tauari, Amazonas State, Brazil, Brazil | -5.9333 | -71.5333 | MPEG 60801 | PQ272270 |
|  | Tefé, Base Petrobras/Urucu, Igarapé Lontra, Amazonas State, Brazil | -4.8667 | -65.1167 | MPEG 57188 | PQ272272 |
|  | 1 km N Rio Napo, 157 km by river NNE Iquitos, Peru | -3.25 | -72.9 | - | MW770933 |
|  | 1 km N Rio Napo, 157 km by river NNE Iquitos, Peru | -3.25 | -72.9 | - | MW770935 |
|  | 1.5 km S Libertad, S. bank Rio Napo, 80 km N Iquitos | -3.27 | -73.08 | - | MW770936 |
|  | 1.5 km S Libertad, S. bank Rio Napo, 80 km N Iquitos, Peru | -3 | -73.33 | - | MW770914 |
|  | 20 km by road NE Tarapoto on road to Yurimaguas, Peru | -6.438559 | -76.303473 | - | MW771040 |
|  | 4 km SE Virgen del Socorro, Heredia, Costa Rica | 10.336536 | -84.155273 | - | MW771028 |
|  | 4 km SE Virgen del Socorro, Heredia, Costa Rica | 10.336536 | -84.155273 | - | MW771034 |
|  | 5 km N Rockstone; E bank Essequibo River, Guyana | 6.033 | -58.550 | - | MW771015 |
|  | 5 km NW Mabura Hill; Btwn Essequibo and Demerara R., Guyana | 5.317 | -58.650 | - | MW770952 |
|  | 5 km NW Mabura Hill; Btwn Essequibo and Demerara R. Guyana | 5.317 | -58.650 | - | MW770953 |
|  | 5KM N Amazonas 85KM NE Iquitos 110M, Peru | -3.42 | -72.58 | - | MW770934 |
|  | 5KM N Amazonas 85KM NE Iquitos 110M, Peru | -3.42 | -72.58 | - | MW770940 |
|  | 5KM N Amazonas 85KM NE Iquitos 110M, Peru | -3.42 | -72.58 | - | MW770944 |
|  | 7km SW Jeberos, Peru | -5.313333 | -76.275556 | - | MW771018 |
|  | 7km SW Jeberos, Peru | -5.313333 | -76.275556 | - | MW771019 |
|  | 7km SW Jeberos, Peru | -5.313333 | -76.275556 | - | MW771020 |
|  | 7km SW Jeberos, Peru | -5.313333 | -76.275556 | - | MW771021 |
|  | Abary River, Guyana | 6.55 | -57.733333 | - | MW770965 |
|  | Amapa, Fazenda Itapoa, Amapá, Brazil | 2.0667 | -50.9333 | - | MW770970 |
|  | Amapa, Fazenda Itapoa, Amapá, Brazil | 2.0667 | -50.9333 | - | MW770971 |
|  | Apoteri, Guyana | 4.283333333 | -58.51666667 | - | MW770962 |
|  | Apoteri, Guyana | 4.283333333 | -58.51666667 | - | MW770968 |
|  | Boven Coesewijne Nature Reserve, Suriname | 5.45 | -55.2 | - | MW770988 |
|  | Boven Coesewijne Nature Reserve, Suriname | 5.45 | -55.2 | - | MW770989 |
|  | Boven Coesewijne Nature Reserve, Suriname | 5.45 | -55.2 | - | MW770990 |
|  | Boven Coesewijne Nature Reserve, Suriname | 5.45 | -55.2 | - | MW770991 |
|  | Boven Coesewijne Nature Reserve, Suriname | 5.45 | -55.2 | - | MW770993 |
|  | Boven Coesewijne Nature Reserve, Suriname | 5.45 | -55.2 | - | MW770994 |
|  | Boven Coesewijne Nature Reserve, Suriname | 5.45 | -55.2 | - | MW770995 |
|  | Boven Coesewijne Nature Reserve, Suriname | 5.45 | -55.2 | - | MW770996 |
|  | Boven Coesewijne Nature Reserve, Suriname | 5.45 | -55.2 | - | MW771001 |
|  | Boven Coesewijne Nature Reserve, Suriname | 5.45 | -55.2 | - | MW771003 |
|  | Boven Coesewijne Nature Reserve, Suriname | 5.45 | -55.2 | - | MW771004 |
|  | Boven Coesewijne Nature Reserve, Suriname | 5.45 | -55.2 | - | MW771005 |
|  | Ca 77 km WNW Contamana, Peru | -7.083333 | -75.65 | - | MW770918 |
|  | Ca 77 km WNW Contamana, Peru | -7.083333 | -75.65 | - | MW770919 |
|  | Ca 77 km WNW Contamana, Peru | -7.083333 | -75.65 | - | MW770920 |
|  | Ca 77 km WNW Contamana, Peru | -7.083333 | -75.65 | - | MW770921 |
|  | Ca 77 km WNW Contamana, Peru | -7.083333 | -75.65 | - | MW770932 |
|  | Ca 86 km SE Juanjui on E bank upper Rio Pauya, Peru | -7.594444 | -75.916111 | - | MW771048 |
|  | Ca 86 km SE Juanjui on E bank upper Rio Pauya, Peru | -7.594444 | -75.916111 | - | MW771049 |
|  | Ca. 86 km SE Juanjui on E bank upper Rio Pauya, Peru | -7.567417 | -75.888889 | - | MW771046 |
|  | Ca. 86 km SE Juanjui on E bank upper Rio Pauya, Peru | -7.566667 | -75.891944 | - | MW771047 |
|  | Caixuana, State of Pará, Brazil | -1.729167 | -51.473611 | - | MW770924 |
|  | Caixuana, State of Pará, Brazil | -1.729167 | -51.473611 | - | MW770925 |
|  | Cerro de La Neblina Base CAMP 140M, Venezuela | 0.833333 | -66.166667 | - | MW770950 |
|  | Cerro de La Neblina Base CAMP 140M, Venezuela | 0.833333 | -66.166667 | - | MW770954 |
|  | Chiriqui Grande then 17km SSW, Palo Seco, Panama | 8.793 | -82.189 | - | MW771030 |
|  | Cord. del Condor; above Chinapinza, Ecuador | -3.950 | -78.550 | - | MW770982 |
|  | Cord. del Condor; above Chinapinza, Ecuador | -3.950 | -78.550 | - | MW771039 |
|  | Dist. Gualaca, Cordillera Central, 4.3 km by road S Lago Fortuna dam, Panama | 8.54435 | -82.302704 | - | MW771031 |
|  | Grand Dégrad, Amapá | 1.977147 | -52.009277 | - | MW770974 |
|  | Grand Dégrad, Amapá | 1.977147 | -52.009277 | - | MW770975 |
|  | Grand Dégrad, Amapá | 1.977147 | -52.009277 | - | MW770976 |
|  | Grand Dégrad, Amapá | 1.977147 | -52.009277 | - | MW770986 |
|  | Grand Dégrad, Amapá | 1.977147 | -52.009277 | - | MW770987 |
|  | Grand Dégrad, Amapá | 1.977147 | -52.009277 | - | MW771006 |
|  | Grand Dégrad, Amapá | 1.977147 | -52.009277 | - | MW771007 |
|  | Grand Dégrad, Amapá | 1.977147 | -52.009277 | - | MW771008 |
|  | Gualaca then 22km NNE, Reserva Forestal Fortuna, Panama | 8.718 | -82.236 | - | MW771027 |
|  | Gualaca then 22km NNE, Reserva Forestal Fortuna, Panama | 8.718 | -82.236 | - | MW771033 |
|  | Gualaca then 22km NNE, Reserva Forestal Fortuna Panama | 8.718 | -82.236 | - | MW771036 |
|  | Ilhéus, Ecoparque de UNA, Brazil | -15.159722 | -39.045 | - | MW770904 |
|  | Ilhéus, Ecoparque de UNA, Brazil | -15.159722 | -39.045 | - | MW770905 |
|  | Ilhéus, Ecoparque de UNA, Brazil | -15.159722 | -39.045 | - | MW770906 |
|  | Iwokrama Reserve; Burro Burro River, ca. 3 miles S Siparuni River, Guyana | 4.750 | -58.050 | - | MW770955 |
|  | Japura, Rio Mapari, Amazonas, Brazil | -2.0497 | -67.2631 | - | MW770947 |
|  | Japura, Rio Mapari, Amazonas, Brazil | -2.0497 | -67.2631 | - | MW770948 |
|  | Japura, Rio Mapari, Amazonas, Brazil | -2.0497 | -67.2631 | - | MW770949 |
|  | Japura, Rio Mapari, Amazonas, Brazil | -2.0497 | -67.2631 | - | MW770958 |
|  | Japura, Rio Mapari, Amazonas, Brazil | -2.0497 | -67.2631 | - | MW770959 |
|  | Japura, Rio Mapari, Amazonas, Brazil | -2.0497 | -67.2631 | - | MW770961 |
|  | Japura, Rio Mapari, Amazonas, Brazil | -2.0497 | -67.2631 | - | MW770966 |
|  | Japura, Rio Mapari, Amazonas, Brazil | -2.0497 | -67.2631 | - | MW770967 |
|  | Japura, Rio Mapari, Amazonas, Brazil | -2.0497 | -67.2631 | - | MW771025 |
|  | Kappel, forest around Kappel Airstrip, Suriname | 3.783 | -56.150002 | - | MW770972 |
|  | Kappel, forest around Kappel Airstrip, Suriname | 3.783 | -56.150002 | - | MW770981 |
|  | Kappel, forest around Kappel Airstrip, Suriname | 3.783 | -56.150002 | - | MW770992 |
|  | Kappel, forest around Kappel Airstrip, Suriname | 3.783 | -56.150002 | - | MW771000 |
|  | Kappel, forest around Kappel Airstrip, Suriname | 3.783 | -56.150002 | - | MW771014 |
|  | Kappel, forest around Kappel Airstrip, Suriname | 3.783 | -56.150002 | - | MW771022 |
|  | Koribeni, Cusco, Peru | -12.7 | -72.875 | - | MW771044 |
|  | Koribeni, Cusco, Peru | -12.7 | -72.875 | - | MW771045 |
|  | Lower Rio Napo region, E bank Rio Yanayacu, ca 90 km N Iquito, Peru | -3.084852778 | -73.13609167 | - | MW770937 |
|  | Lower Rio Napo region, E bank Rio Yanayacu, ca 90 km N Iquitos, Peru | -3.084852778 | -73.13609167 | - | MW770938 |
|  | Lower Urubamba, Centro Pucani, 10o40'S 73o32'W, Peru | -10.66 | -73.532 | - | MW770917 |
|  | Maraa, Lago Cumapi, Amazonas State, Brazil | -1.73 | -65.8792 | - | MW770957 |
|  | Mata da Pancada Grande, Reserva Michelin, Bahia State, Brazil | -13.784389 | -39.173861 | - | MW771057 |
|  | Mata do Pacangê, Reserva Michelin, Bahia State, Brazil | -13.784389 | -39.173861 | - | MW770908 |
|  | Morawhanna | 8.25 | -59.73333333 | - | MW770960 |
|  | Munic. Manaus; km 34 ZF-3, Faz. Esteio, ca 80 km N. Manaus, Amazonas State, Brazil | -2 | -59 | - | MW770946 |
|  | Munic. Manaus; km 34 ZF-3, Faz. Esteio, ca 80 km N. Manaus, Amazonas State, Brazil | -2 | -59 | - | MW770951 |
|  | Munic. Manaus; km 34 ZF-3, Faz. Esteio, ca 80 km N. Manaus, Amazonas State, Brazil | -2 | -59 | - | MW770964 |
|  | Municipio Alta Floresta, upper Rio Teles Pires-Rio Cristalino, Mato Grosso State, Brazil | -9.886111 | -56.087222 | - | MW770939 |
|  | Municipio Alta Floresta, upper Rio Teles Pires-Rio Cristalino, Mato Grosso State, Brazil | -9.886111 | -56.087222 | - | MW770941 |
|  | Municipio Alta Floresta, upper Rio Teles Pires-Rio Cristalino, Mato Grosso State, Brazil | -9.886111 | -56.087222 | - | MW770942 |
|  | Municipio Alta Floresta, upper Rio Teles Pires-Rio Cristalino, Mato Grosso State, Brazil | -9.886111 | -56.087222 | - | MW770943 |
|  | Parque Estadual Paulo César Vinha, Espírito Santo, Brazil | -20.620861 | -40.428 | - | MW770900 |
|  | Parque Estadual Paulo César Vinha, Espírito Santo, Brazil | -20.620861 | -40.428 | - | MW770907 |
|  | Pasoburco; KM 57 on Hollin-Loreto Road, Ecuador | -0.691435, | -77.309375 | - | MW771038 |
|  | Pasoburco; KM 57 on Hollin-Loreto Road, Ecuador | -0.691435, | -77.309375 | - | MW771037 |
|  | Poço das Antas Biological Reserve, Rio de Janeiro, Brazil | -22.3 | -42.1 | - | MW771056 |
|  | Poço das Antas Biological Reserve, Rio de Janeiro, Brazil | -22.3 | -42.1 | - | MW771058 |
|  | Portel, FLONA de Caxiuanã, Plot PPBIO, Pará State, Brazil | -1.95 | -51.6 | - | MW770926 |
|  | Portel, FLONA de Caxiuanã, Plot PPBIO, Pará State, Brazil | -1.95 | -51.6 | - | MW770927 |
|  | Portel, FLONA de Caxiuanã, Plot PPBIO, Pará State, Brazil | -1.95 | -51.6 | - | MW770928 |
|  | Portel, FLONA de Caxiuanã, Plot PPBIO, Pará State, Brazil | -1.95 | -51.6 | - | MW770929 |
|  | Portel, FLONA de Caxiuanã, Plot PPBIO, Pará State, Brazil | -1.95 | -51.6 | - | MW770930 |
|  | Portel, FLONA de Caxiuanã, Plot PPBIO, Pará State, Brazil | -1.95 | -51.6 | - | MW770931 |
|  | Puellas; Km 41 on Villa Rica - Puerto Bermudez highway, Peru | -10.67 | -75.1 | - | MW771043 |
|  | Restinga de Jurubatiba, Rio de Janeiro State, Brazil | -22.275278 | -41.663611 | - | MW770901 |
|  | Restinga de Jurubatiba, Rio de Janeiro State, Brazil | -22.275278 | -41.663611 | - | MW770902 |
|  | Restinga de Jurubatiba, Rio de Janeiro State, Brazil | -22.275278 | -41.663611 | - | MW770909 |
|  | Rio Amazonas, ca 10 km SSW mouth Rio Napo on E. bank Quebrada Vainilla, Peru | -3.538962 | -72.738726 | - | MW770922 |
|  | Rio Amazonas, ca 10 km SSW mouth Rio Napo on E. bank Quebrada Vainilla, Peru | -3.538962 | -72.738726 | - | MW770923 |
|  | S Rio Amazonas, CA 10KM SSW Rio Napo, Loreto, Peru | -3.538962 | -72.738726 | - | MW770910 |
|  | San Antonio, Junín, Peru | -11.472 | -74.791 | - | MW771041 |
|  | San Antonio, Junín, Peru | -11.472 | -74.791 | - | MW771042 |
|  | San Jacinto, Loreto, Peru | -2.529408 | -75.729683 | - | MW770911 |
|  | San Jacinto, Loreto, Peru | -2.529408 | -75.729683 | - | MW770912 |
|  | San Jacinto, Loreto, Peru | -2.529408 | -75.729683 | - | MW770913 |
|  | San Jacinto, Loreto, Peru | -2.529408 | -75.729683 | - | MW770915_ |
|  | San Jacinto, Loreto, Peru | -2.529408 | -75.729683 | - | MW771024 |
|  | Santa Terezinha, Serra da Jiboia, Bahia State, Brazil | -12.51 | -39.28 | - | MW770903 |
|  | Surumatra, Potaro-Siparuni, Guyana | 4.333333333 | -58.85 | - | MW770945 |
|  | Surumatra, Potaro-Siparuni, Guyana | 4.333333333 | -58.85 | - | MW770963 |
|  | Tafelberg, Heliodoxa Camp, Sipaliwini Distrikt, Suriname | 3.89835 | -56.1621 | - | MW770956 |
|  | Tafelberg, Heliodoxa Camp, Sipaliwini Distrikt, Suriname | 3.89835 | -56.1621 | - | MW770977 |
|  | Tafelberg, Heliodoxa Camp, Sipaliwini Distrikt, Suriname | 3.89835 | -56.1621 | - | MW770983 |
|  | Tafelberg, Heliodoxa Camp, Sipaliwini Distrikt, Suriname | 3.89835 | -56.1621 | - | MW770984 |
|  | Tafelberg, Heliodoxa Camp, Sipaliwini Distrikt, Suriname | 3.89835 | -56.1621 | - | MW770985 |
|  | Tafelberg, Heliodoxa Camp, Sipaliwini Distrikt, Suriname | 3.89835 | -56.1621 | - | MW770997 |
|  | Tafelberg, Heliodoxa Camp, Sipaliwini Distrikt, Suriname | 3.89835 | -56.1621 | - | MW770998 |
|  | Tafelberg, Heliodoxa Camp, Sipaliwini Distrikt, Suriname | 3.89835 | -56.1621 | - | MW770999 |
|  | Tafelberg, Heliodoxa Camp, Sipaliwini Distrikt, Suriname | 3.89835 | -56.1621 | - | MW771009 |
|  | Tafelberg, Heliodoxa Camp, Sipaliwini Distrikt, Suriname | 3.89835 | -56.1621 | - | MW771010 |
|  | Tafelberg, Heliodoxa Camp, Sipaliwini Distrikt, Suriname | 3.89835 | -56.1621 | - | MW771012 |
|  | Tafelberg, Heliodoxa Camp, Sipaliwini Distrikt, Suriname | 3.89835 | -56.1621 | - | MW771016 |
|  | Tafelberg, south side of Arrowhead Basin, Sipaliwini Distrikt, Suriname | 3.9022 | -56.1711 | - | MW770969 |
|  | Tafelberg, Tafelberg north rim at Augustus Creek Waterfall, Sipaliwini Distrikt, Suriname | 3.93 | -56.1883 | - | MW770973 |
|  | Tafelberg, Tafelberg north rim at Augustus Creek Waterfall, Sipaliwini Distrikt, Suriname | 3.93 | -56.1883 | - | MW770978 |
|  | Tafelberg, Tafelberg north rim at Augustus Creek Waterfall, Sipaliwini Distrikt, Suriname | 3.93 | -56.1883 | - | MW771017 |
|  | Tafelberg, Tafelberg north rim at Augustus Creek Waterfall, Sipaliwini Distrikt, Suriname | 3.93 | -56.1883 | - | MW771026 |
|  | Tafelberg, upper Cayman Creek, Sipaliwini Distrikt, Suriname | 3.9017 | -56.18 | - | MW770979 |
|  | Tafelberg, upper Cayman Creek, Sipaliwini Distrikt, Suriname | 3.9017 | -56.18 | - | MW770980 |
|  | Tafelberg, upper Cayman Creek, Sipaliwini Distrikt, Suriname | 3.9017 | -56.18 | - | MW771002 |
|  | Tucurrique; Reserva Biológica El Copal, Cartago Province, Costa Rica | 9.783889 | -83.751667 | - | MW771029 |
|  | Tucurrique; Reserva Biológica El Copal, Cartago Province, Costa Rica | 9.783889 | -83.751667 | - | MW771032 |
|  | Tucurrique; Reserva Biológica El Copal,Cartago Province, Costa Rica | 9.783889 | -83.751667 | - | MW771035 |
|  | W. bank Rio Shesha, 65 km ENE Pucallpa, Ucayali Department, Peru | -8.174042 | -73.993375 | - | MW770916 |
|  | Wilhelmina Mountains, ridgetop 8 km N of Juliana Top, Sipaliwini Distrikt, Suriname | 3.7533 | -56.5217 | - | MW771011 |
|  | Wilhelmina Mountains, ridgetop 8 km N of Juliana Top, Sipaliwini Distrikt, Suriname | 3.7533 | -56.5217 | - | MW771013 |
| *Ceratopipra chloromeros* | - | - | - | - | KF228551 |
| *Ceratopipra erythrocephala* | - | - | - | - | KF228553 |
| *Ceratopipra mentalis* | - | - | - | - | DQ294535 |
| *Ceratopipra cornuta* | - | - | - | - | KF228552 |
| *Machaeropterus deliciosus* | - | - | - | - | GU985501 |
| *Lepidothrix*  *coronata* | - | - | - | - | MW771055 |
| *Heterocercus*  *linteatus* | - | - | - | - | GU985500 |
| *Manacus manacus* | - | - | - | - | GU985504 |

Tissue type: b = blood; l=liver-

UERJ - Universidade Estadual de Feira de Santana

MPEG – Museu Paraense Emílio Gueldi

MZFS – Museu de Zoologia de Feira de Santana

MZUSP – Museu de Zoologia da Universidade de São Paulo

FMNH - Field Museum Natural History

INPA – Instituto National de Pesquisas da Amazônia

AMNH - American Museum of Natural History

**File S1.** **Materials and Methods**

*DNA sampling and sequencing*

Samples were obtained through loans from Museu Emílio Goeldi (MPEG), Museu de Zoologia da Universidade de São Paulo (MZUSP), Museu de Zoologia da Universidade Estadual de Feira de Santana (MZFS), the Field Museum of Natural History (FMNH), the American Museum of Natural History (AMNH) and Instituto Nacional de Pesquisas da Amazônia (INPA) and complemented with targeted field trips to eight Atlantic Forest localities (Table S1).

Molecular laboratory methods for DNA sequencing followed standard protocols. Whole genome DNA extraction was performed with a Qiagen DNeasy Blood & Tissue Extraction Kit (Qiagen, Valencia, CA). The entire mtDNA gene NADH dehydrogenase subunit II (ND2; 1041 bp) was amplified with four primer combinations: L5216 (Sorenson *et al*., 1999) and H5766 (Sorenson *et al*., 1999) or H6313 (Sorenson *et al*., 1999), L5602 (Ribas *et al*., 2005) and H6312 (Cicero & Johnson, 2001), and L5204 (Cicero & Johnson, 2001) and H6315 (Kirchman *et al*., 2001). Amplification was performed with 4 µL 5X PCR buffer, 2 µL MgCl (25mM), 2 µL dNTP, 1.3 µL of primers (10 µM), 0.2 µL HotStart Taq polymerase, 8.2 µL H20 to 20 µL, and 1 µL of template. PCR conditions included a 2 min denaturation step (94°C), five cycles of denaturation at 94°C (30 s) followed by annealing at 58°C (30 s) and extension at 70°C (90 s), ten similar cycles at 56°C annealing temperature, ten cycles at 54°C annealing temperature, 15 similar cycles at 52°C annealing temperature, followed by a final extension step (120s). PCR products were analyzed by gel electrophoresis, cleaned up with vacuum manifold (Eppendorf 5 Prime), and re-suspended in 100uL of DNA free water. Sequencing reactions used a BigDye Terminator Kit (Applied Biosystems, USA). Cycle sequencing reaction products were precipitated with a 70% ethanol solution (100 µL), which was added to each product and centrifuged for 45 minutes at 27^o^C and 4000 rpm. Water (35 µL) was then added to each solution; sequencing reactions were run on a 3730XL DNA Analyzer (Applied Biosystems, USA), using instrument protocol 50cm POP7BDB-1.

**References**

Cicero, C. & Johnson, N.K. (2001). Higher-level phylogeny of new world Vireos (Aves: Vireonidae) based on sequences of multiple mitochondrial DNA genes. *Molecular Phylogenetics and Evolution*, **20(1)**: 27–40.

Kirchman J.J., Shannon, J.H., Goodman, S.M. & Bates, J.M. (2001). Phylogeny and systematics of ground rollers (Brachypteraciidae) of Madagascar. *The Auk*, **118:** 849–863.

Ribas, C.C., Gaban-Lima, R., Miyaki, C.Y. & Cracraft, J. (2005). Historical biogeography and diversification within the Neotropical parrot genus *Pionopsitta* (Aves: Psittacidae). *Journal of Biogeography*, **32:** 1409-1427.

Sorenson, M.D., Ast, J.C., Dimcheff, D.E., Yuri, T. & Mindell, D.P. (1999). Primers for a PCR-based approach to mitochondrial genome sequencing in birds and other vertebrates. Molecular *Phylogenetics and Evolution*, **12:** 105–114.

**Table S2.** Occurrence data for *Ceratopipra rubrocapilla* and *Dixiphia pipra* that were used for Species Distribution Modelling.

| **Specie** | **Country** | **State** | **Locality** | **Latitude Dec** | **Longitude Dec** |
| --- | --- | --- | --- | --- | --- |
| *Ceratopipra rubrocapilla* | Brasil | Alagoas | Ibateguara, Engenho Coimbra, Usina Serra Grande | -8.96667 | -35.85 |
| *Ceratopipra rubrocapilla* | Brazil | Alagoas | Murici Biological Forest, 59km NW of Maceio. | -9.29595 | -35.945 |
| *Ceratopipra rubrocapilla* | Brazil | Alagoas | Ibateguara | -8.9835 | -35.8428 |
| *Ceratopipra rubrocapilla* | Brasil | Pernambuco | Timbauba | -7.6 | -35.3667 |
| *Ceratopipra rubrocapilla* | Brasil | Pernambuco | Serra do Espelho | -7.81667 | -34.85 |
| *Ceratopipra rubrocapilla* | Brasil | Pernambuco | Mata do Estado | -7.58333 | -35.5 |
| *Ceratopipra rubrocapilla* | Brasil | Pernambuco | Macambira | -8.6 | -36.4333 |
| *Ceratopipra rubrocapilla* | Brazil | Pernambuco | Saltinho Biological Reserve | -8.65808 | -35.1181 |
| *Ceratopipra rubrocapilla* | Brazil | Pernambuco | Parque Dois Irmãos, Recife | -8.05 | -34.8667 |
| *Ceratopipra rubrocapilla* | Brazil | Pernambuco | Mata de Aldeia, Camaragipe | -8.01667 | -34.9667 |
| *Ceratopipra rubrocapilla* | Brazil | Pernambuco | Engenho Cachoeira Linda, Barreiros | -8.81667 | -35.4667 |
| *Ceratopipra rubrocapilla* | Brazil | Sergipe | Santa Luzia do Itanhy, Crato forest ; Aracaju mudflats | -11.3402 | -37.4487 |
| *Ceratopipra rubrocapilla* | Brazil | Espírito Santo | Fazenda Cupido e Refúgio, Linhares | -19.05 | -39.9667 |
| *Ceratopipra rubrocapilla* | Brazil | Espírito Santo | Reserva Natural da Vale do Rio Doce, Linhares | -19.151 | -40.0259 |
| *Ceratopipra rubrocapilla* | Brazil | Espirito Santo | Pau Gigante | -19.8333 | -40.3667 |
| *Ceratopipra rubrocapilla* | Brazil | Rio de Janeiro | União Biological Reserve | -22.4167 | -42.0333 |
| *Ceratopipra rubrocapilla* | Brazil | Rio de Janeiro | Poço das Antas Biological Reserve | -22.5 | -42.1667 |
| *Ceratopipra rubrocapilla* | Brasil | Bahia | RPPN Serra Bonita, Fazenda Paris, Camacan | -15.4167 | -39.5333 |
| *Ceratopipra rubrocapilla* | Brazil | Bahia | Restinga Trancoso | -16.5333 | -39.1 |
| *Ceratopipra rubrocapilla* | Brazil | Bahia | Igrapiúna, Reserva da Michelin | -13.8333 | -39.1667 |
| *Ceratopipra rubrocapilla* | Brazil | Bahia | Santa Terezinha, Serra da Jiboia | -12.85 | -39.4667 |
| *Ceratopipra rubrocapilla* | Brazil | Bahia | Ecoparque de UNA, Ilhéus | -15.15 | -39.7 |
| *Ceratopipra rubrocapilla* | Brazil | Bahia | Serra da Jibóia, Fazenda Jequitibá, Elísio Medrado | -12.8667 | -39.4667 |
| *Ceratopipra rubrocapilla* | Brazil | Bahia | Jequie to Salvador, stop in forest | -13.9807 | -39.8502 |
| *Ceratopipra rubrocapilla* | Brazil | Bahia | Fazenda Ouro (Wana) | -14.9 | -39.1 |
| *Ceratopipra rubrocapilla* | Brazil | Bahia | c. 3 Km W of Fazenda (Aruana), near Una | -15.1833 | -39.2833 |
| *Ceratopipra rubrocapilla* | Brazil | Bahia | Michelin Forest Reserve, Ituberá | -13.8333 | -39.2333 |
| *Ceratopipra rubrocapilla* | Brazil | Bahia | Reserva Capitão, Itacaré | -14.3167 | -39 |
| *Ceratopipra rubrocapilla* | Brazil | Amazonas | Manicoré, Rodovia do Estanho, km 136 | -8.68333 | -61.4 |
| *Ceratopipra rubrocapilla* | Brazil | Amazonas | Município de Humaitá, Terra Indígena Parintintin, Aldeia Pupunha | -7.46667 | -62.9333 |
| *Ceratopipra rubrocapilla* | Brazil | Amazonas | RDS Cujubim, ca 390 km SW Jutaí | -5.21667 | -68.3167 |
| *Ceratopipra rubrocapilla* | Brazil | Amazonas | RDS Cujubim, margem esquerda do Rio Jutaí | -5.63333 | -69.1667 |
| *Ceratopipra rubrocapilla* | Brazil | Amazonas | Humaitá, Território Indígena Ipixuna, Aldeia Canavial, Miriti | -6.55 | -62.05 |
| *Ceratopipra rubrocapilla* | Brazil | Amazonas | Coari, Rio Urucu, Trilha do Papagaio | -4.85 | -65.0667 |
| *Ceratopipra rubrocapilla* | Brazil | Amazonas | Maués, Flona do Pau Rosa, Comunidade Caiaué | -4.01667 | -58.4333 |
| *Ceratopipra rubrocapilla* | Brazil | Amazonas | Humaitá, margem esquerda Rio Madeira, Ipixuna | -7.51667 | -63.3333 |
| *Ceratopipra rubrocapilla* | Brazil | Amazonas | Estirão do Equador, Atalaia do Norte | -4.51667 | -71.6 |
| *Ceratopipra rubrocapilla* | Brazil | Amazonas | Amazon River | -7.6667 | -65.7667 |
| *Ceratopipra rubrocapilla* | Brazil | Amazonas | Barra de São Manoel | -7.3464 | -58.1597 |
| *Ceratopipra rubrocapilla* | Brazil | Amazonas | Pousada on left bank Rio Juruena | -7.64228 | -58.2366 |
| *Ceratopipra rubrocapilla* | Brazil | Pará | Município de Juruti, Base Capiranga, Igarapé Mutum | -2.6 | -56.1833 |
| *Ceratopipra rubrocapilla* | Brazil | Pará | Rio Xingu, margem direita, Senador José Porfírio | -3.51667 | -51.7167 |
| *Ceratopipra rubrocapilla* | Brazil | Pará | Belterra, Flona do Tapajós, Santarém/Cuiabá, BR 163 Km 117 | -3.35 | -54.9333 |
| *Ceratopipra rubrocapilla* | Brazil | Pará | Novo Progresso, Base Aeronaútica Serra do Cachimbo, Torre II | -9.26667 | -54.9333 |
| *Ceratopipra rubrocapilla* | Brazil | Pará | Jacareacanga, Transamazônica, ponte sobre o Rio Igarapé Preto | -5.9 | -57.6833 |
| *Ceratopipra rubrocapilla* | Brazil | Pará | Novo Progresso, Flona Jamanxim, Rio Jamanxim, margem esquerda | -6.53333 | -55.65 |
| *Ceratopipra rubrocapilla* | Brazil | Pará | 20 km SW de Novo Progresso | -7.18333 | -55.4833 |
| *Ceratopipra rubrocapilla* | Brazil | Pará | Itaituba, FLONA Amanã, Garimpo JMS, margem direita Igarapé Porquinho | -5.1 | -57.5333 |
| *Ceratopipra rubrocapilla* | Brazil | Pará | Santarém, Retiro | -2.38333 | -55.7833 |
| *Ceratopipra rubrocapilla* | Brazil | Pará | Portel, FLONA do Caxiuanã, Plot PPBIO | -1.95 | -51.6 |
| *Ceratopipra rubrocapilla* | Brazil | Pará | Placas, Assentamento Comunidade Fortaleza | -3.78333 | -54.9333 |
| *Ceratopipra rubrocapilla* | Brazil | Pará | Jacareacanga, FLONA do Crepori, Rio das Tropas, Cotovelo | -6.51667 | -57.4333 |
| *Ceratopipra rubrocapilla* | Brazil | Pará | Jacareacanga, Igarapé do Rato | -5.4 | -56.9167 |
| *Ceratopipra rubrocapilla* | Brazil | Pará | Jacareacanga, Aproeste | -6.48333 | -58.15 |
| *Ceratopipra rubrocapilla* | Brazil | Pará | Aveiro, Rio Mamuru | -3.31667 | -56.35 |
| *Ceratopipra rubrocapilla* | Brazil | Pará | Santarém, RESEX Tapajós-Arapiuns, Alto-Mentai | -2.78333 | -55.6 |
| *Ceratopipra rubrocapilla* | Brazil | Para | Vila Braga | -4.4167 | -56.2833 |
| *Ceratopipra rubrocapilla* | Brazil | Para | Itaituba | -4.2833 | -55.9833 |
| *Ceratopipra rubrocapilla* | Brazil | Para | Portel, FLONA do Caxiuana, Plot PPBIO | -1.95 | -51.6 |
| *Ceratopipra rubrocapilla* | Brazil | Para | Santarem | -2.4333 | -54.7 |
| *Ceratopipra rubrocapilla* | Brazil | Para | Benevides | -1.3667 | -48.25 |
| *Ceratopipra rubrocapilla* | Brazil | Para | Vigia | -0.8 | -48.1333 |
| *Ceratopipra rubrocapilla* | Brazil | Rondônia | Município de Ji-Paraná, Igarapé Lurdes, Aldeia Gaviões | -10.4333 | -61.65 |
| *Ceratopipra rubrocapilla* | Brazil | Rondônia | Rio Ouro Preto, margem esquerda, Guajará-Mirim, Reserva Biológica Ouro Preto | -10.8333 | -64.75 |
| *Ceratopipra rubrocapilla* | Brazil | Rondônia | Machadinho D'Oeste, margem direita Rio Jiparaná | -8.9 | -62 |
| *Ceratopipra rubrocapilla* | Brazil | Rondônia | Fortaleza do Abunã | -9.76667 | -65.5167 |
| *Ceratopipra rubrocapilla* | Brazil | Rondônia | Pousada Ecológica Rancho Grande | -10.2979 | -62.8667 |
| *Ceratopipra rubrocapilla* | Brazil | Rondônia | Rio Verde to Lago do Cuniã | -8.34624 | -63.41 |
| *Ceratopipra rubrocapilla* | Brazil | Mato Grosso | Canarana, Fazenda Tanguro | -12.8833 | -52.3667 |
| *Ceratopipra rubrocapilla* | Brazil | Mato Grosso | Paranaíta, Rio Teles Pires | -9.41667 | -56.75 |
| *Ceratopipra rubrocapilla* | Brazil | Mato Grosso | Paranaíta, margem direita Rio Teles Pires, Sete Quedas | -9.3 | -57.5833 |
| *Ceratopipra rubrocapilla* | Brazil | Mato Grosso | Paranaíta, margem direita Rio Teles Pires, Sete Quedas | -9.31667 | -56.7833 |
| *Ceratopipra rubrocapilla* | Brazil | Mato Grosso | Querência, Fazenda Tanguro | -12.8833 | -52.3667 |
| *Ceratopipra rubrocapilla* | Brazil | Mato Grosso | Xingu Refúgio Amazonico | -12.1441 | -54.1046 |
| *Ceratopipra rubrocapilla* | Brazil | Mato Grosso | Rio Cristalino | -9.44906 | -56.3599 |
| *Ceratopipra rubrocapilla* | Brazil | Mato Grosso | Serra dos Caiabis, Alta Floresta | -10.75 | -56.75 |
| *Ceratopipra rubrocapilla* | Brazil | Acre | Tarauacá, Br 364 km 40 Rio Liberdade, margem direita | -7.88333 | -71.65 |
| *Ceratopipra rubrocapilla* | Brazil | Acre | Porto Acre, Reserva Humaitá | -9.75 | -67.6667 |
| *Ceratopipra rubrocapilla* | Brazil | Acre | Senador Guiomard, Br 364 km 80, Ramal Oco do Mundo km 16 | -9.83333 | -67.1667 |
| *Ceratopipra rubrocapilla* | Brazil | Acre | Porto Walter, Igarapé Cruzeiro do Vale, Colônia Dois Portos | -8.33333 | -72.6 |
| *Ceratopipra rubrocapilla* | Peru | Madre de Dios | 0 km SW from Puerto Maldonado; Tambopata Reserve | -12.88 | -69.28 |
| *Ceratopipra rubrocapilla* | Peru | Madre de Dios | 0 km from Pampas del Heath | -12.7 | -68.8 |
| *Ceratopipra rubrocapilla* | Peru | Madre de Dios | Puerto Maldonado (Puesto de Control Enahuipa) | -12.517 | -68.7 |
| *Ceratopipra rubrocapilla* | Peru | Loreto | Amazon Research Center | -4.33152 | -73.2373 |
| *Ceratopipra rubrocapilla* | Peru | Loreto | Rio Yavari | -4.2 | -70.2333 |
| *Ceratopipra rubrocapilla* | Peru | Loreto | Santa Cecilia | -3.7667 | -73.25 |
| *Ceratopipra rubrocapilla* | Peru | Loreto | Yanamono | -3.46202 | -72.7958 |
| *Ceratopipra rubrocapilla* | Peru | Madre de Dios | Cuzco Amazonico Reserve, 14 km E Puerto Maldonado | -12.55 | -69.05 |
| *Ceratopipra rubrocapilla* | Peru | Madre de Dios | 1 km SW from Puerto Maldonado; Tambopata Reserve | -4.2893 | -72.2226 |
| *Ceratopipra rubrocapilla* | Bolivia | Santa Cruz | Noel Kempff Mercado National Park, Huanchaca Dos | -14.2667 | -60.8667 |
| *Ceratopipra rubrocapilla* | Bolivia | La Paz | 0 km from Puesto Heath; Madidi National Park | -14.55 | -67.72 |
| *Ceratopipra rubrocapilla* | Bolivia | Santa Cruz | Estancia Caparu, Puesto Lagunitas | -14.8094 | -61.1746 |
| *Ceratopipra rubrocapilla* | Bolivia | Santa Cruz | 0 km from Noel Kempff Mercado National Park; Campamento Los Fierros | -14.55 | -60.93 |
| *Ceratopipra rubrocapilla* | Bolivia | Santa Cruz | Fin del camino de los fierros | -14.5529 | -60.7986 |
| *Ceratopipra rubrocapilla* | Bolivia | La Paz | 0 km from Puesto Ganadero; Madidi National Park, county Franz Tamayo Province | -13.27 | -68.57 |
| *Ceratopipra rubrocapilla* | Bolivia | La Paz | Puerto Moscoso, Parque Nacional Madidi | -13.633 | -68.733 |
| *Ceratopipra rubrocapilla* | Bolivia | Santa Cruz | Flor de Oro, Parue Nacional Noel Kempff | -13.535 | -61.008 |
| *Ceratopipra rubrocapilla* | Bolivia | Santa Cruz | Lago Caiman, Parue Nacional Noel Kempff | -13.6 | -60.915 |
| *Ceratopipra rubrocapilla* | Bolivia | Pando | Concesion Industria Madereira Pando (IMAPA) | -11.0504 | -69.2386 |
| *Ceratopipra rubrocapilla* | Bolivia | Pando | 0 km SW from: Cobija; Camino Mueden | -11.08 | -68.89 |
| *Ceratopipra rubrocapilla* | Bolivia | Pando | Site 5, Main Camp on Rio Negro | -9.867 | -65.7 |
| *Pseudopipra pipra* | Brazil | Rio de Janeiro | União Biological Reserve | -22.4167 | -42.0333 |
| *Pseudopipra pipra* | Brazil | Rio de Janeiro | Poço das Antas Biological Reserve | -22.5 | -42.1667 |
| *Pseudopipra pipra* | Brazil | Rio de Janeiro | Fazenda São Lázaro, Restinga de Jurubatiba | -22.2667 | -41.65 |
| *Pseudopipra pipra* | Brazil | Espírito Santo | Parque Estadual Paulo César Vinha | -20.6167 | -40.4167 |
| *Pseudopipra pipra* | Brazil | Espírito Santo | Reserva Natural da Vale do Rio Doce | -19.1167 | -39.95 |
| *Pseudopipra pipra* | Brazil | Bahia | Santa Terezinha, Serra da Jibóia | -12.85 | -39.4667 |
| *Pseudopipra pipra* | Brazil | Bahia | Ecoparque de UNA, Ilhéus | -15.15 | -39.0333 |
| *Pseudopipra pipra* | Brazil | Bahia | Reserva Capitão, Itacaré | -14.3167 | -39 |
| *Pseudopipra pipra* | Brazil | Bahia | Fazenda Ouro (Wana) | -14.9 | -39.1 |
| *Pseudopipra pipra* | Brazil | Bahia | c. 3 Km W of Fazenda Aruana, near Una | -15.1833 | -39.2833 |
| *Pseudopipra pipra* | Brazil | Bahia | RPPN Estação Veracel, Porto Seguro | -16.333 | -39.1333 |
| *Pseudopipra pipra* | Brazil | Amazonas | Tefé, Base Petrobras/Urucu, Igarapé Lontra | -4.8667 | -65.1167 |
| *Pseudopipra pipra* | Brazil | Amazonas | Município de Coari, Base Petrobrás/Urucu, Igarapé Onça | -4.8667 | -65.3 |
| *Pseudopipra pipra* | Brazil | Amazonas | Novo Airão, Igarapé-Açu | -2.85 | -60.85 |
| *Pseudopipra pipra* | Brazil | Amazonas | Rio Cuiuni, margem direita, Barcelos | -0.7833 | -63.15 |
| *Pseudopipra pipra* | Brazil | Amazonas | Japurá, Rio Mapari | -2.0333 | -67.2833 |
| *Pseudopipra pipra* | Brazil | Amazonas | Maraã, Lago Cumapi | -1.7167 | -65.8667 |
| *Pseudopipra pipra* | Brazil | Amazonas | Oriximiná | -1.5335 | -54.9094 |
| *Pseudopipra pipra* | Brazil | Amazonas | Rio Aracá, Barcelos | -0.4167 | -60.9333 |
| *Pseudopipra pipra* | Brazil | Amazonas | RDS Cujubim, margem W Baixo Rio Mutum | -4.9333 | -68.1667 |
| *Pseudopipra pipra* | Brazil | Amazonas | Urucará, linhão Tucuruí, Manaus | -2.3833 | -57.6333 |
| *Pseudopipra pipra* | Brazil | Amazonas | Careiro, Br 319 km 158, Tupana Lodge | -4.0833 | -60.65 |
| *Pseudopipra pipra* | Brazil | Amazonas | Estirão do Equador, Atalaia do Norte | -4.5167 | -71.6 |
| *Pseudopipra pipra* | Brazil | Amazonas | Amazon River | -7.6667 | -65.7667 |
| *Pseudopipra pipra* | Brazil | Amazonas | Novo Airao | -2.6167 | -60.9333 |
| *Pseudopipra pipra* | Brazil | Amazonas | Balbina, Presidente Figueiredo, Amazonas | -1.9333 | -59.4167 |
| *Pseudopipra pipra* | Brazil | Amazonas | left bank Rio Bararati | -7.5028 | -58.258 |
| *Pseudopipra pipra* | Brazil | Amazonas | Rio Negro Day 3 | -2.1309 | -61.1004 |
| *Pseudopipra pipra* | Brazil | Amazonas | Rio Negro, above Manaus | -2.9019 | -60.5649 |
| *Pseudopipra pipra* | Brazil | Amazonas | Presidente Figueiredo | -2.0320 | -60.0197 |
| *Pseudopipra pipra* | Brazil | Amazonas | Parque Nacional do Jaú | -1.8835 | -61.7375 |
| *Pseudopipra pipra* | Brazil | Amazonas | Parque Nacional do Jaú, chabascal trail 5km from entrance | -1.9208 | -61.4573 |
| *Pseudopipra pipra* | Brazil | Amazonas | Left bank Rio Bararati | -7.5028 | -58.2580 |
| *Pseudopipra pipra* | Brazil | Amazonas | Rio Araca, Barcelos | -0.4167 | -62.9333 |
| *Pseudopipra pipra* | Brazil | Amazonas | Manaus, Agropecuário Da Suframa, km 33, Zf-3 | -2.63 | -59.83 |
| *Pseudopipra pipra* | Brazil | Amazonas | Agropecuario da Suframa, Manaus | -2.0 | -59.7772 |
| *Pseudopipra pipra* | Brazil | Amazonas | Manaus | -2.596 | -59.9785 |
| *Pseudopipra pipra* | Brazil | Pará | Novo Progresso, Base Aeronaútica Serra do Cachimbo, Torre II | -9.2667 | -54.9333 |
| *Pseudopipra pipra* | Brazil | Pará | Município de Santa Bárbara, Gunma | -1.1833 | -48.2833 |
| *Pseudopipra pipra* | Brazil | Pará | Sítio do Waldir, Breves, Marajó | -1.55 | -50.3833 |
| *Pseudopipra pipra* | Brazil | Pará | Portel, FLONA do Caxiuanã, Plot PPBIO | -1.95 | -51.6 |
| *Pseudopipra pipra* | Brazil | Pará | Flota de Faro, ca 70 km NW de Faro | -1.7 | -57.2 |
| *Pseudopipra pipra* | Brazil | Pará | Alenquer, ESEC Grão-Pará | -0.15 | -55.18333333 |
| *Pseudopipra pipra* | Brazil | Pará | Igarapé Engano, Flona Jamanxim, Novo Progresso | -7.7 | -55.65 |
| *Pseudopipra pipra* | Brazil | Pará | Oriximiná, ESEC Grão Pará | 1.2833 | -58.6833 |
| *Pseudopipra pipra* | Brazil | Pará | Flota do Trombetas, Óbidos | -0.95 | -55.5167 |
| *Pseudopipra pipra* | Brazil | Pará | Flota do Paru, Almeirim | -0.9333 | -53.2333 |
| *Pseudopipra pipra* | Brazil | Pará | REBIO Maicuru, Almeirim | 0.8167 | -53.9167 |
| *Pseudopipra pipra* | Brazil | Pará | ESEC Grão-Pará, Óbidos | 0.6167 | -55.7167 |
| *Pseudopipra pipra* | Brazil | Pará | FLONA do Crepori, Rio das Tropas, Cotovelo, Jacareacanga | -6.5167 | -57.4333 |
| *Pseudopipra pipra* | Brazil | Pará | FLONA do Trairão, Trairão | -4.5333 | -55.2 |
| *Pseudopipra pipra* | Brazil | Pará | Rio Xingu, Vila Maracanã, Faro | -2.0667 | -56.6167 |
| *Pseudopipra pipra* | Brazil | Pará | Tomé-Açu, margem direita Rio Tocantins | -2.5 | -47.9833 |
| *Pseudopipra pipra* | Brazil | Pará | Itaituba, Km 85 Transgarimpeira | -6.9833 | -56.1667 |
| *Pseudopipra pipra* | Brazil | Pará | Comunidade Casinha, Lago Sapucuá, Oriximiná | -1.75 | -56.2167 |
| *Pseudopipra pipra* | Brazil | Pará | Pousada Rio Azul | -13.8333 | -39.2333 |
| *Pseudopipra pipra* | Brazil | Pará | Novo Progresso | -7.15 | -55.4833 |
| *Pseudopipra pipra* | Brazil | Pará | Flona Caxiuanã | -1.8 | -50.7667 |
| *Pseudopipra pipra* | Brazil | Pará | Bacia 100, Paragominas | -2.742 | -47.8681 |
| *Pseudopipra pipra* | Brazil | Pará | Caxiuanã | -1.7335 | -51.4556 |
| *Pseudopipra pipra* | Brazil | Pará | Beinafica (Bemfica) | -1.3 | -48.3 |
| *Pseudopipra pipra* | Brazil | Amapá | Rio Amapari, Parque Nacional Montanhas do Tumucumaque | 1.6 | -52.4833 |
| *Pseudopipra pipra* | Brazil | Amapá | Rio Mapaoni, Parque Nacional Montanhas do Tumucumaque | 2.1833 | -54.5833 |
| *Pseudopipra pipra* | Brazil | Amapá | Rio Anotaie, Parque Nacional Montanhas do Tumucumaque | 3.2 | -52.1 |
| *Pseudopipra pipra* | Brazil | Amapá | Rio Mutum, Parque Nacional Montanhas do Tumucumaque | 1.3833 | -51.9167 |
| *Pseudopipra pipra* | Brazil | Amapá | Rio Anacuí, Parque Nacional Montanhas do Tumucumaque | 1.8333 | -52.7333 |
| *Pseudopipra pipra* | Brazil | Acre | Tarauacá, Br 364 km 40 Rio Liberdade, margem direita | -7.8833 | -71.65 |
| *Pseudopipra pipra* | Brazil | Acre | Tarauacá, Floresta Estadual do Mogno, Br 364 km 6 Rio Tauari | -5.9333 | -71.5333 |
| *Pseudopipra pipra* | Brazil | Acre | Tarauacá, Floresta Estadual Rio Gregório, próximo ponte Rio Acuraua | -8.0667 | -71.1667 |
| *Pseudopipra pipra* | Brazil | Acre | Feijó, Rio Envira, Novo Porto, Foz do Igarapé Paraná do Ouro | -8.45 | -70.55 |
| *Pseudopipra pipra* | Brazil | Acre | Mâncio Lima, Estrada do Barão Comunidade São Domingos | -7.55 | -72.9833 |
| *Pseudopipra pipra* | Brazil | Acre | Feijó, Baixo Rio Jurupari, Humaitá | -7.95 | -69.9333 |
| *Pseudopipra pipra* | Brazil | Acre | Feijó, Rio Jurupari, margem esquerda, Novo Oriente | -8.2167 | -69.85 |
| *Pseudopipra pipra* | Brazil | Acre | Jordão | -9.2 | -71.85 |
| *Pseudopipra pipra* | Brazil | Mato Grosso | Alta Floresta | -9.8667 | -56.0833 |
| *Pseudopipra pipra* | Brazil | Mato Grosso | Serra dos Caiabis, Alta Floresta | -10.75 | -56.75 |
| *Pseudopipra pipra* | Brazil | Mato Grosso | Rio Cristalino | -9.4490 | -56.3379 |
| *Pseudopipra pipra* | Brazil | Mato Grosso | Rio Teles Pires, Ilha Cristalino | -9.6332 | -55.9393 |
| *Pseudopipra pipra* | Peru | Cusco | ca. Alto Materiato | -12.7 | -72.875 |
| *Pseudopipra pipra* | Peru | Junin | Along Rio Satipo | -11.472 | -74.791 |
| *Pseudopipra pipra* | Peru | Loreto | Sabalillo | -3.35 | -72.2833 |
| *Pseudopipra pipra* | Peru | Loreto | Tierra Blanca | -4.2667 | -77.2333 |
| *Pseudopipra pipra* | Peru | Cusco | Llactahuaman, Quillabamba | -12.865 | -73.513 |
| *Pseudopipra pipra* | Peru | Ucayali | upper Ucayali valley - Sapani | -10.7096 | -73.8822 |
| *Pseudopipra pipra* | Peru | Ucayali | upper Ucayali valley - Cohengua Stunted Forest | -10.4130 | -73.6780 |
| *Pseudopipra pipra* | Peru | Huánuco | Tingo Maria | -9.2919 | -75.9760 |
| *Pseudopipra pipra* | Peru | Ucayali | Junin Pablo | -8.9056 | -74.2653 |
| *Pseudopipra pipra* | Peru | Ucayali | Contamana Hills | -7.1987 | -74.9453 |
| *Pseudopipra pipra* | Peru | San Martin | Rio Verde camp | -6.7188 | -77.4268 |
| *Pseudopipra pipra* | Peru | San Martin | Quebrada Mishquyacu | -6.0748 | -76.9790 |
| *Pseudopipra pipra* | Peru | Amazonas | Pampa del Burro | -5.6406 | -77.9470 |
| *Pseudopipra pipra* | Peru | Loreto | Lagunas | -5.2396 | -75.6616 |
| *Pseudopipra pipra* | Peru | Loreto | Centro Investigaciones Jenaro Herrera | -4.8997 | -73.6507 |
| *Pseudopipra pipra* | Peru | Loreto | Allpahuayo-Mishana Reserve | -4.1444 | -73.4937 |
| *Pseudopipra pipra* | Peru |  | Upper Rio Comainas | -3.917 | -78.433 |
| *Pseudopipra pipra* | Peru | Loreto | Explornapo Lodge | -3.2579 | -72.91745 |
| *Pseudopipra pipra* | Peru | Loreto | Piedras Camp (Rio Algodoncillo) | -2.7928 | -72.9170 |
| *Pseudopipra pipra* | Peru | Loreto | Campamento Choro | -2.6106 | -71.4859 |
| *Pseudopipra pipra* | Peru | La Libertad | Above Utcubamba on trail to Ongon | -8.28 | -77.3 |
| *Pseudopipra pipra* | Peru | Pasco | Prov. Oxapampa; Distrito Puerto Bermudez; Comunidad San Juan | -10.5043 | -74.8081 |
| *Pseudopipra pipra* | Peru | Amazonas | Quebrada Huacabamba | -6.5917 | -77.5533 |
| *Pseudopipra pipra* | Ecuador | Orellana | Shiripuno Amazon Lodge, Huaorani reserve, border of Orellana and Plastaza | -1.1 | -76.7167 |
| *Pseudopipra pipra* | Equador | Orellana | Yuturi Lodge | -0.5333 | -76.3833 |
| *Pseudopipra pipra* | Equador | Orellana | Yasuni Research Station, Parque Nacional Yasuni | -0.6667 | -76.3833 |
| *Pseudopipra pipra* | Ecuador | Orellana | Yuturi Lodge | -0.5333 | -76.0333 |
| *Pseudopipra pipra* | Ecuador | Zamora-Chinchipe | Tepui Trail, Cabañas Yankuam, Zamora-Chinchipe | -4.25 | -78.6833 |
| *Pseudopipra pipra* | Ecuador | Morona-Santiago | Kapawi Lodge | -2.5680 | -76.7277 |
| *Pseudopipra pipra* | Ecuador | Zamora-Chinchipe | Podocarpus National Park, Bombuscaro entrada | -4.1095 | -78.9660 |
| *Pseudopipra pipra* | Ecuador | Napo | Gareno Lodge | -1.0357 | -77.3972 |
| *Pseudopipra pipra* | Ecuador | Napo | loreto road chonta yacu bridge, ecuador | -0.6949 | -77.6871 |
| *Pseudopipra pipra* | Ecuador | Orellana | Yasuni National Park | -0.9602 | -76.0041 |
| *Pseudopipra pipra* | Ecuador | Napo | San Jose Nuevo | -0.4333 | -75.3333 |
| *Pseudopipra pipra* | Ecuador | Napo | Napo Lodge | -0.6033 | -75.9158 |
| *Pseudopipra pipra* | Ecuador | Sucumbios | Rio Verde | 0.2372 | -77.5764 |
| *Pseudopipra pipra* | Ecuador | Pastaza | Rio Rutuno | -1.9166 | -77.2333 |
| *Pseudopipra pipra* | Colombia | Valle del Cauca | Alto Anchicaya | 3.5 | -76.5833 |
| *Pseudopipra pipra* | Colombia | Valle del Cauca | Alto Anchicaya, corregimiento El Danubio, Valle | 3.38333 | -76.7833 |
| *Pseudopipra pipra* | Colombia | Meta | Cubarral, Vereda Aguas Claras, Serrania de Aguas Claras | 3.8161 | -73.9195 |
| *Pseudopipra pipra* | Colombia | Boyacá | Boyaca, Corregimiento de Paez, Vereda de El Tunjo | 5.0833 | -73.05 |
| *Pseudopipra pipra* | Colombia | Meta | Plateau, Mt. Macarena | 2.75 | -73.9164 |
| *Pseudopipra pipra* | Colombia | Antioquia | Salazar, Bodega Vieja, Trocha a Aguadenos, cuenca del Rio Riachon | 6.9716 | -75.0568 |
| *Pseudopipra pipra* | Colombia | Vichada | Selva de Mataven, Cano Cajaro | 4.5589 | -68.1975 |
| *Pseudopipra pipra* | Colombia | Vichada | Selva de Matavén, Río Orinoco | 4.6092 | -67.8644 |
| *Pseudopipra pipra* | Colombia | Antioquia | Salgar | 5.97 | -75.98 |
| *Pseudopipra pipra* | Colombia | Antioquia | Anorí | 7.07 | -75.15 |
| *Pseudopipra pipra* | Colombia | Santander | Río Negro. Fca. San Isidro (Pablo Contreras) | 7.1228 | -72.245 |
| *Pseudopipra pipra* | Colombia | Antioquia | Arrierito Antiqueno Reserve | 7.0212 | -75.1403 |
| *Pseudopipra pipra* | Colombia | Caquetá | Inspeccion de policía La Esmeralda. Alto Rio Yurayaco | 1.3486 | -76.1031 |
| *Pseudopipra pipra* | Colombia | Guaviare | Cano Cocuy, Cerro Moyano, Río Inírida | 2.1764 | -71.1828 |
| *Pseudopipra pipra* | Colombia | Nariño | Cuenca alta de los Rios Rumiyaco y Rancheria. Territorio Kofan. | 0.4683 | -77.2855 |
| *Pseudopipra pipra* | Colombia | Caquetá | Rio Mesay | 0.2422 | -72.9375 |
| *Pseudopipra pipra* | Venezuela | Amazonas | Dpto. Rio Negro, vicinity of Cerro de la Neblina base camp on Rio Mawarinuma | 0.8333 | -66.1667 |
| *Pseudopipra pipra* | Venezuela | Amazonas | Mavaca Tapriapeco Base Camp; Rio Mavaca | 2.03 | -65.12 |
| *Pseudopipra pipra* | Venezuela | Amazonas | Camturama | 5.2183 | -67.7952 |
| *Pseudopipra pipra* | Venezuela | Bolívar | Rio Grande, Imataca Forest Reserve | 8.0449 | -61.6429 |
| *Pseudopipra pipra* | Venezuela | Bolívar | Upata | 8.17 | -61.75 |
| *Pseudopipra pipra* | Venezuela | Bolívar | Las Claritas--Capuchinbird Road | 6.18267 | -61.4106 |
| *Pseudopipra pipra* | Venezuela | Amazonas | Subregion Orinoco | 3.971 | -67.111 |
| *Pseudopipra pipra* | Venezuela | Bolívar | Subregion Ventuari | 4.193 | -66.749 |
| *Pseudopipra pipra* | Venezuela | Bolívary | Upata | 6.5 | -66.5 |
| *Pseudopipra pipra* | Suriname | Sipaliwini | Palumeu Jungle Lodge, Suriname | 3.1542 | -55.7529 |
| *Pseudopipra pipra* | Suriname | Sipaliwini | Suriname - Palumeu, river, Kwepipan trail, Abrase trail | 3.70804 | -54.9193 |
| *Pseudopipra pipra* | Suriname | Heliodoxa Camp | Sipaliwini Distrikt | 3.8984 | -56.1621 |
| *Pseudopipra pipra* | Suriname | Sipaliwini Distrikt | Ridgetop 8 km N of Juliana Top | 3.7533 | -56.5217 |
| *Pseudopipra pipra* | Suriname | Para Distrikt, | Boven Coesewijne Nature Reserve | 5.45 | -55.2 |
| *Pseudopipra pipra* | Suriname | Neger Kreek | Marowijne Distrikt | 5.5833 | -54.2 |
| *Pseudopipra pipra* | French Guiana | Cayenne | Cayene river | 4.9333 | -52.333 |
| *Pseudopipra pipra* | French Guiana | Roura | Oyak River | 4.6584 | -52.3387 |
| *Pseudopipra pipra* | French Guiana | Iracoubo | Saint-Laurent-du-Maroni | 5.4833 | -54.05 |
| *Pseudopipra pipra* | French Guiana | Iracoubo | Fleuve Mana | 5.15 | -53.75 |
| *Pseudopipra pipra* | French Guiana | Saul | Saul | 3.6172 | -53.2086 |
| *Pseudopipra pipra* | French Guiana | Angoulême | Angoulême | 5.4103 | -53.6552 |
| *Pseudopipra pipra* | Guyana | Georgetown | Iwokrama Forest, White Sand Forest | 4.3667 | -58.85 |
| *Pseudopipra pipra* | Guyana | Georgetown | Iwokrama Forest, Atta Harpy Eagle, nest trail | 4.2333 | -58.9 |
| *Pseudopipra pipra* | Guyana | Georgetown | Essequibo; Waruma River | 2.9 | -58.9333 |
| *Pseudopipra pipra* | Guyana | Georgetown | Essequibo Islands-West Demerara | 2.0835 | -59.2495 |
| *Pseudopipra pipra* | Guyana | Potaro-Siparuni | Iwokrama Field Station | 4.6827 | -58.6950 |
| *Pseudopipra pipra* | Guyana | Cuyuni-Mazaruni | Kamarang River | 5.8686 | -60.6093 |
| *Pseudopipra pipra* | Guyana | Upper Demerara-Berbice | Shiribina Creek area | 5.5333 | -58.1833 |
| *Pseudopipra pipra* | Guyana | Mahaica-Berbice | Taurakuli, Abary River | 5.9553 | -57.7958 |
| *Pseudopipra pipra* | Guyana | Guyana | Shanklands Area | 6.4381 | -58.6024 |
| *Pseudopipra pipra* | Panama | Chiriquí | Gualaca, 22 km NNE; Reserva Forestal Fortuna | 8.7167 | -82.2333 |
| *Pseudopipra pipra* | Costa Rica | Cartago | Rancho Naturalista | 9.8167 | -83.55 |
| *Pseudopipra pipra* | Costa Rica | Alajuela | Celeste Mountain Lodge | 10.7139 | -85.0441 |
| *Pseudopipra pipra* | Costa Rica | San Jose | Quebrada Gonzalez | 10.1394 | -83.9555 |
| *Pseudopipra pipra* | Costa Rica | Cartago | Guayabo | 9.95 | -83.65 |
